# Electronic Health Record Interventions to Reduce Risk of Hospital Readmissions: A Systematic Review and Meta-Analysis

**DOI:** 10.1001/jamanetworkopen.2025.21785

**Published:** 2025-07-17

**Authors:** Badal S. B. Pattar, Abigail Ackroyd, Emir Sevinc, Taylor Hecker, Keila Turino Miranda, Caitlin McClurg, Kyle Weekes, Matthew T. James, Neesh Pannu, Pietro Ravani, Paul E. Ronksley, Sofia B. Ahmed, Tyrone G. Harrison

**Affiliations:** 1 Department of Medicine, Cumming School of Medicine, University of Calgary, Calgary, Alberta, Canada; 2 Libin Cardiovascular Institute, University of Calgary, Calgary, Alberta, Canada; 3 O’Brien Institute for Public Health, University of Calgary, Calgary, Alberta, Canada; 4 Cardiovascular Health and Autonomic Regulation Laboratory, Department of Kinesiology and Physical Education, McGill University, Montreal, Quebec, Canada; 5 Libraries and Cultural Resources, University of Calgary, Calgary, Alberta, Canada; 6 Department of Community Health Sciences, University of Calgary, Calgary, Alberta, Canada; 7 Department of Medicine, University of Alberta, Edmonton, Alberta, Canada

## Abstract

**Importance:**

Hospital readmissions are associated with significant health care costs and poor patient outcomes. Despite the rapid adoption of electronic health record (EHR) systems, the use of EHR-based interventions to reduce the risk of hospital readmissions is unknown.

**Objective:**

To systematically review and estimate the association of EHR-based interventions vs controls with preventing 30-day all-cause hospital readmissions as tested in randomized clinical trials (RCTs).

**Data Sources:**

Ovid MEDLINE, Ovid Embase, CINAHL, the Cochrane Central Register of Controlled Trials, and ClinicalTrials.gov were searched from database inception to July 5, 2024, using text words with analogous terms within concept areas of “randomized controlled trial,” “hospitalized adults,” and “readmissions.”

**Study Selection:**

RCTs were included if they evaluated the effect of EHR-based interventions on hospital readmissions compared with a control arm without an EHR-embedded component. Studies were excluded if they involved nonhospitalized, pediatric, obstetric, or psychiatric populations or did not report readmission outcomes. Results were reported in accordance with the Preferred Reporting Items for Systematic Reviews and Meta-Analyses reporting guideline.

**Data Extraction and Synthesis:**

Data were extracted independently by 3 reviewers in duplicate. A random-effects model was used to pool data, and the quality of studies was assessed using the Cochrane Risk of Bias tool. Heterogeneity was quantified using the *I*^2^ statistic and explored with prespecified subgroup analyses and univariable meta-regression by population demographics, intervention complexity, and publication year.

**Main Outcomes and Measures:**

The primary outcome was 30-day all-cause hospital readmission, and other readmission outcomes (eg, unplanned readmissions and readmissions at 3, 6, 12, and 24 months) were examined as secondary outcomes.

**Results:**

A total of 116 RCTs involving 204 523 participants (weighted mean [SD] males, 56% [16%]; weighted mean [SD] age, 68 [9] years) were included, with telemonitoring (76 studies [66%]) being the most common EHR-based intervention component followed by case management (45 studies [39%]) and medication reconciliation (33 [28%]). EHR-based interventions were associated with a statistically significant reduction in 30-day all-cause readmissions (OR, 0.83 [95% CI, 0.70-0.99]; *I^2^* = 82%; τ = 0.44 [95% CI, 0.30-0.62]; prediction interval [PI], 0.34-2.06) and 90-day all-cause readmissions (OR, 0.72 [95% CI, 0.54-0.96]; *I^2^* = 78%; τ = 0.34 [95% CI, 0.19-1.00]; PI, 0.33-1.55) compared with control arms.

**Conclusions and Relevance:**

In this systematic review and meta-analysis of RCTs, the use of EHR-based interventions was associated with a reduction in 30-day and 90-day hospital readmissions. Future research should examine additional components of EHR interventions to understand and account for remaining gaps in effectiveness.

## Key Points

**Question:**

Are electronic health record (EHR)–based interventions associated with reduced risk of hospital readmissions?

**Findings:**

This systematic review and meta-analysis of 116 randomized clinical trials with 204 523 participants found that EHR-based interventions were associated with reduced risk of 30-day and 90-day all-cause readmission by 17% and 28%, respectively.

**Meaning:**

These findings highlight the potential for leveraging EHR systems to improve patient and health system outcomes, but further research is required to understand which components of EHR interventions drive their effectiveness and for whom.

## Teaser

This systematic review and meta-analysis investigates whether electronic health record (EHR)–based interventions are associated with reduced hospital readmissions vs controls with no EHR-embedded component.

## Introduction

Readmission after hospitalization is common, has implications for the quantity and quality of life for patients and the allocation of health care resources, and is used as a measure of hospital quality. Worldwide, hospitals are operating at critical capacity, which has been further exacerbated by aging,^1^ prolonged survival among patients with complex diseases,^2^ and challenges, such as the COVID-19 pandemic.^3^ All-cause readmission rates among all US health care system payers have previously been estimated to be 14% within 30 days.^4^ Not only do readmissions place significant economic burden on health care systems,^5,6^ but they are also associated with more than 2 times higher risk of short- and long-term mortality 2 years after hospitalization.^7^ While some readmissions are anticipated, a significant portion are unplanned, with approximately 30% being potentially avoidable.^8^ As such, reducing readmissions has become a priority^9^ that may lower costs, enhance quality of care, and improve clinical outcomes and the patient experience.^10^

Many interventions have been proposed to reduce hospital readmission risk. A previous systematic review by Leppin et al^11^ investigated a variety of interventions to reduce readmissions and found that overall, interventions were associated with a reduction in 30-day all-cause readmission with consistent findings across patient subgroups. Since that time, the use of electronic health records (EHRs) has emerged as a potentially effective approach to support interventions aimed at reducing readmissions. The incorporation of EHR technology within clinical settings may improve care quality, decrease occurrence of medical errors, and lead to enhancements in patient-level indicators of care appropriateness.^12^ As EHR technology has become increasingly integrated into clinical care around the world, many interventions are delivered using this technology.

Despite the increasing use of EHRs with the enhanced capability to conduct embedded randomized clinical trials (RCTs), at this juncture, we do not know which EHR-based interventions are effective at reducing readmissions and how they should be deployed. In this systematic review and meta-analysis, we aimed to summarize and synthesize the current evidence on the efficacy of EHR-delivered interventions in reducing the risk of 30-day all-cause hospital readmission.

## Methods

This systematic review and meta-analysis was reported in accordance with the Preferred Reporting Items for Systematic Reviews and Meta-Analyses (PRISMA) reporting guideline.^13^ The review protocol was registered in PROSPERO.

### Data Sources and Searches

In collaboration with a research librarian, we designed and performed a comprehensive search of multiple databases on June 17, 2023, including Ovid MEDLINE, Ovid Embase, CINAHL, the Cochrane Central Register of Controlled Trials, and ClinicalTrials.gov, from inception using text words with analogous terms within concept areas of “randomized controlled trial,” “hospitalized adults,” and “readmissions.” Relevant articles were also hand-searched and included. This search was updated on July 5, 2024. Database-specific search strategies are provided in eTable 1 in Supplement 1.

### Eligibility Criteria and Study Selection

Eligible studies were RCTs conducted in hospitalized adult populations with the aim of reducing hospital readmissions. The interventions had to be delivered entirely or in part using EHR technology, which was defined as a real-time health data record accessible in immediate and secured fashion to authorized users.^14^ Control arms had no intervention with an EHR-embedded component. As this level of detail was often not included in the titles and abstracts of relevant articles, we did not require this criterion to be met during the first screening stage; this criterion was applied at the full-text phase. The review excluded studies involving pediatric, obstetric, or psychiatric populations as well as studies that did not report readmission outcomes. There were no language restrictions, and studies in non-English languages were translated using an online translation tool previously validated in systematic reviews.^15,16^ Conference abstracts meeting eligibility criteria were included in this study but were excluded from the risk-of-bias assessment.

Using Covidence (Cochrane Technology), 3 reviewers (B.S.B.P., A.A., and K.W.) independently screened titles and abstracts in duplicate. A calibration exercise was performed whereby the first 100 decisions were reviewed to ensure each reviewer was appropriately applying the eligibility criteria. Studies deemed eligible by 1 or more reviewers were propagated to the full-text phase of the review. At this stage, 4 reviewers (B.S.B.P., A.A., T.H., and K.T.M.) independently reviewed full-text articles in duplicate for inclusion. Disagreements between the reviewers were resolved by a fifth reviewer (T.G.H.).

### Data Extraction and Risk of Bias

Data from eligible studies were extracted independently by 1 of 3 reviewers (B.S.B.P., A.A., and T.H.), and a second reviewer reviewed each extraction for accuracy. A fourth reviewer (T.G.H.) resolved any discrepancies found in the data extraction. We extracted data on participant and intervention characteristics, the primary outcome of 30-day all-cause readmission, and secondary outcomes, including 30-day risk of unplanned readmissions and a composite end point of all-cause readmission and deaths. Data on these readmission outcome types at 90 days and at 6, 12, and 24 months were also extracted. Interventions were described using the activity-based coding framework for discharge interventions used by Leppin et al^11^ (eTable 2 in Supplement 1). The EHR-embedded component of each intervention was described using a framework developed by Gagnon et al^17^ that included 4 categories that described the system, function, timing, and facilitation of the intervention (eTable 2 in Supplement 1). Outcomes of interest were reported as odds ratios (ORs) or other effect estimates, including hazard ratios and risk ratios, with accompanying 95% CIs. In the event that studies did not report ORs, numbers of outcome events and participants in each trial arm were extracted, where reported, and used to estimate ORs.

One of 3 reviewers (B.S.B.P., A.A., and T.H.) independently assessed the risk of bias of included studies using the revised Cochrane Risk of Bias tool for RCTs.^18^ The 5 domains were assessed as low risk, some risk, or high risk, and the overall risk score was determined by the highest risk-of-bias level in any of the domains. A second reviewer reviewed these results, and any discrepancies were resolved by a fourth reviewer (T.G.H.).

### Statistical Analysis

Meta-analyses were conducted by pooling ORs for each trial using a random-effects model per the Mantel-Haenszel method.^19^ Results were visualized with forest plots presenting individual and pooled effect estimates along with 95% CIs and prediction intervals (PIs). *Q*, *I*^2^, and τ statistics were used to investigate between-study heterogeneity. Subgroup and univariable meta-regression analyses were performed on our primary outcome of interest using variables determined a priori, including mean participant age, proportion of each sex in the study population, year of publication, number of participants, target population, attending service, EHR brand or vendor being used, specific intervention component, and number of components. Demographic data were summarized with means (SDs) where appropriate, with weighting of each study’s reported measure based on overall proportion within the number of participants included in this review. Evidence of publication bias was examined using funnel plot analysis and the Egger test. All analyses were performed using R Studio 2022.12.0, build 353 (RStudio, PBC), and R, version 4.2.3 (R Project for Statistical Computing). Statistical significance was defined as a 2-sided *P* < .05.

## Results

### Study Selection

**Figure 1.**
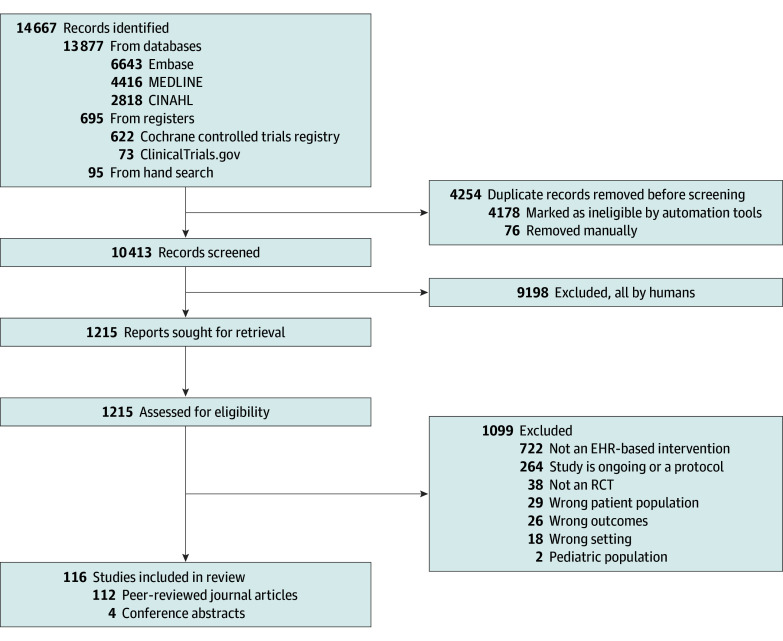
PRISMA Flow Diagram of Process for Study Inclusion EHR, electronic health record; RCT, randomized clinical trial.

Our search identified 10 413 nonduplicate articles, from which 9198 articles were excluded during title and abstract screening (Figure 1). Of the 1215 full texts screened, the most common reason for exclusion was no EHR-based intervention being reported (722 [59%]). Overall, 116 studies^20,21,22,23,24,25,26,27,28,29,30,31,32,33,34,35,36,37,38,39,40,41,42,43,44,45,46,47,48,49,50,51,52,53,54,55,56,57,58,59,60,61,62,63,64,65,66,67,68,69,70,71,72,73,74,75,76,77,78,79,80,81,82,83,84,85,86,87,88,89,90,91,92,93,94,95,96,97,98,99,100,101,102,103,104,105,106,107,108,109,110,111,112,113,114,115,116,117,118,119,120,121,122,123,124,125,126,127,128,129,130,131,132,133,134,135^ involving 204 523 participants met inclusion criteria, including 112 peer-reviewed journal articles^20,21,22,23,24,25,26,27,28,29,30,31,32,33,34,35,36,37,38,39,40,41,42,43,44,45,46,48,49,50,51,52,53,54,55,56,57,58,59,60,61,62,63,64,65,66,67,68,70,71,72,73,74,75,76,77,78,79,80,81,82,83,84,85,86,87,89,91,92,93,94,95,96,97,98,99,100,101,102,103,104,105,106,107,108,109,110,111,112,113,114,115,116,117,118,119,120,121,122,123,124,125,126,127,128,129,130,131,132,133,134,135^ and 4 conference abstracts^47,69,88,90^ (eAppendix in Supplement 1).

### Study and Participant Characteristics

Characteristics of the included studies and participants are described in Table 1. Studies were published between January 1, 2002, and December 31, 2024, with 48 studies (41%) published between January 1, 2016, and December 31, 2020, and 56 (48%) conducted in the US, and had a mean (SD) follow-up time of 0.5 (0.6) years. In total, 207 652 participants were included across all studies, who had a weighted mean (SD) age of 68 (9) years. The weighted mean (SD) percentage of females was 44% (16%), of males was 56% (16%), and of participants with hypertension was 59% (20%). The most common admission diagnosis was heart failure (38 studies [33%]),^20,21,23,26,29,31,32,33,38,41,42,43,48,50,55,56,57,64,69,70,73,75,77,84,85,89,94,101,106,107,108,109,116,119,123,131,132,135^ with cardiology being the most common attending service (48 studies [41%]).^20,21,23,26,29,31,32,33,37,38,41,42,43,48,50,54,55,56,57,64,67,68,69,70,72,73,75,76,77,83,84,85,89,94,101,106,107,108,109,110,119,125,126,128,129,131,132,134^ Furthermore, most intervention components included telemonitoring (76 [66%])^20,22,23,26,27,29,31,35,38,40,41,42,43,46,47,48,49,50,51,54,55,56,57,63,64,66,67,68,69,70,71,73,75,76,77,78,79,80,81,83,84,85,87,88,89,90,91,93,94,95,97,98,99,101,104,106,107,108,109,111,112,114,115,117,118,119,121,122,125,126,128,129,131,133,134,135^ and communication with a health care professional (86 [74%]).^20,22,23,25,26,30,31,32,34,35,38,39,41,42,43,45,47,48,49,50,51,55,56,57,58,59,61,63,64,65,66,67,68,69,70,73,74,75,77,78,79,80,81,82,83,84,85,86,87,88,89,91,93,94,96,97,98,99,100,101,102,103,104,105,106,107,108,109,111,112,114,115,116,118,119,120,121,123,125,128,129,130,131,132,134,135^ Nearly half of interventions had a duration greater than 30 days (44 [38%])^22,26,27,28,31,32,35,38,40,41,43,48,50,54,56,57,66,67,72,77,78,81,85,87,89,97,101,104,106,107,108,112,115,117,118,119,121,123,125,126,128,129,133,135^ and were partially supported by a health care professional, including the patient and/or caregiver as part of the intervention. Ninety-one studies (78%) included at least 2 components, in which telemonitoring (59 studies [65%]),^22,23,26,29,31,35,38,40,42,43,46,47,48,49,50,51,54,55,56,57,63,67,68,70,71,73,75,76,77,78,79,80,81,83,87,89,93,94,97,98,101,104,106,108,109,112,114,117,118,121,122,125,126,128,129,131,133,134,135^ case management (45 studies [49%]),^22,28,29,31,32,34,42,44,49,50,51,54,55,57,59,65,68,70,71,73,74,75,77,78,79,81,83,86,89,91,93,98,100,101,102,106,109,114,117,118,120,122,125,126,127^ and telephone follow-up (35 studies [38%])^24,25,26,28,31,32,34,37,40,42,43,47,48,55,56,58,61,73,74,77,93,94,100,101,103,104,106,109,114,121,123,129,130,132,134^ were the most common (eTable 3 in Supplement 1).

**Table 1.** Characteristics of Included Studies

Characteristic^a^	Studies (N = 116)^b^
Total study participants included, No.	204 523
Sample size, mean (range)	1762 (10-144 868)
Publication year	
<2010	14 (12)
2010-2015	18 (16)
2016-2020	48 (41)
>2020	36 (31)
Location of publication	
Canada	6 (5)
China	5 (4)
Denmark	4 (3)
England	13 (11)
France	3 (3)
Japan	3 (3)
Netherlands	6 (5)
Spain	3 (3)
Switzerland	3 (3)
US	56 (48)
Other	14 (12)
Age, mean (SD), y	68 (9)
Participant sex, mean (SD) % of total participants	
Females	44 (16)
Males	56 (16)
Participants with hypertension, mean (SD) % of total participants	59 (20)
Participants with diabetes, mean (SD) % of total participants	34 (17)
Smoked tobacco products, mean (SD) % of total participants	29 (26)
Admission diagnosis, mean (SD) % of total participants	
Acute coronary syndrome	11 (10)
Acute exacerbation of COPD	9 (8)
Cancer	2 (2)
Heart failure	38 (33)
Inflammatory bowel disease	1 (1)
Multiple diagnoses	21 (18)
Organ transplant	2 (2)
Sepsis	2 (2)
Surgery	8 (7)
Unspecific or not reported	22 (19)
Attending service	
Cardiology	48 (41)
Internal medicine	3 (3)
Surgery	10 (8)
Multiple services	12 (10)
Other or not reported	43 (37)
Intervention duration, d	
<1	4 (3)
1-7	2 (2)
8-30	21 (18)
>30	44 (38)
Unknown or unclear	45 (39)
Intervention component^c^	
Case management	45 (39)
Clinician continuity	12 (10)
Discharge planning	17 (15)
Follow-up scheduled	6 (5)
Home visits	4 (3)
Making requisites	5 (4)
Medication reconciliation	33 (28)
Patient-centered discharge instructions	8 (7)
Patient education	32 (28)
Patient hotline	8 (7)
Rehabilitation intervention	2 (2)
Self-management	24 (21)
Streamlining	3 (3)
Telemonitoring	76 (66)
Telephone follow-up	35 (30)
Timely follow-up	10 (9)
Timely PCP communication	5 (4)
Other	14 (12)
Intervention system^c^	
Computer (eg, software)	26 (22)
Electronic health record	116 (100)
Electronic messaging (eg, email)	16 (14)
Internet or website	12 (10)
Mobile app	19 (16)
Robot	1 (1)
Telehealth (eg, telemedicine, telepsychiatry)	68 (59)
Other	3 (2)
Function of EHR-embedded component^c^	
Communication with health care professional	86 (74)
Communication with peers	5 (4)
Other psychotherapy	1 (1)
Prompts and alerts (eg, decision support)	55 (47)
Health care professional monitoring	75 (65)
Self-monitoring	16 (14)
Screening (eg, decision support)	29 (25)
Transmission of information (eg, 1-way communication)	77 (66)
Other	6 (5)
Intervention timing	
Asynchronous	3 (3)
Synchronous	113 (97)
Intervention facilitation	
Entirely supported by health care professional	44 (38)
Partially supported by health care professional	69 (59)
Self-administered	3 (3)
EHR brand	
Epic systems	8 (7)
Multiple	6 (5)
Other	53 (46)
Not reported	49 (42)
Follow-up time, mean (SD), y	0.5 (0.6)
Reported all-cause readmission	
No	49 (42)
Yes	67 (58)
Reported unplanned readmission	
No	110 (95)
Yes	6 (5)
Reported composite outcome	
No	106 (91)
Yes	10 (9)

Abbreviations: COPD, chronic obstructive pulmonary disease; EHR, electronic health record; PCP, primary care professional.

^a^ Missing data: age, n = 22; females, n = 5; hypertension, n = 86; diabetes, n = 73; and smoked tobacco products, n = 101.

^b^ Data are presented as weighted number (percentage) of studies or, where indicated, as weighted means (SDs).

^c^ Results may not add to 100% or the total number of studies given multiple options being reported in a single study.

Over half of the studies (67 [58%]) reported all-cause readmissions,^20,21,22,24,25,28,29,31,33,34,36,37,41,43,44,45,46,47,48,49,51,52,55,56,59,60,61,62,64,65,66,67,68,74,78,80,83,84,86,89,93,94,95,96,98,100,101,104,105,106,109,111,112,113,114,116,119,120,121,123,124,126,127,128,130,133,135^ whereas reporting of unplanned readmissions (6 studies [5%])^38,70,71,102,103,115^ and our composite outcome (10 [9%])^22,41,49,74,84,94,105,109,114,116^ was minimal. Individual study characteristics are summarized in eTable 4 in Supplement 1.

### Meta-Analysis

**Figure 2.**
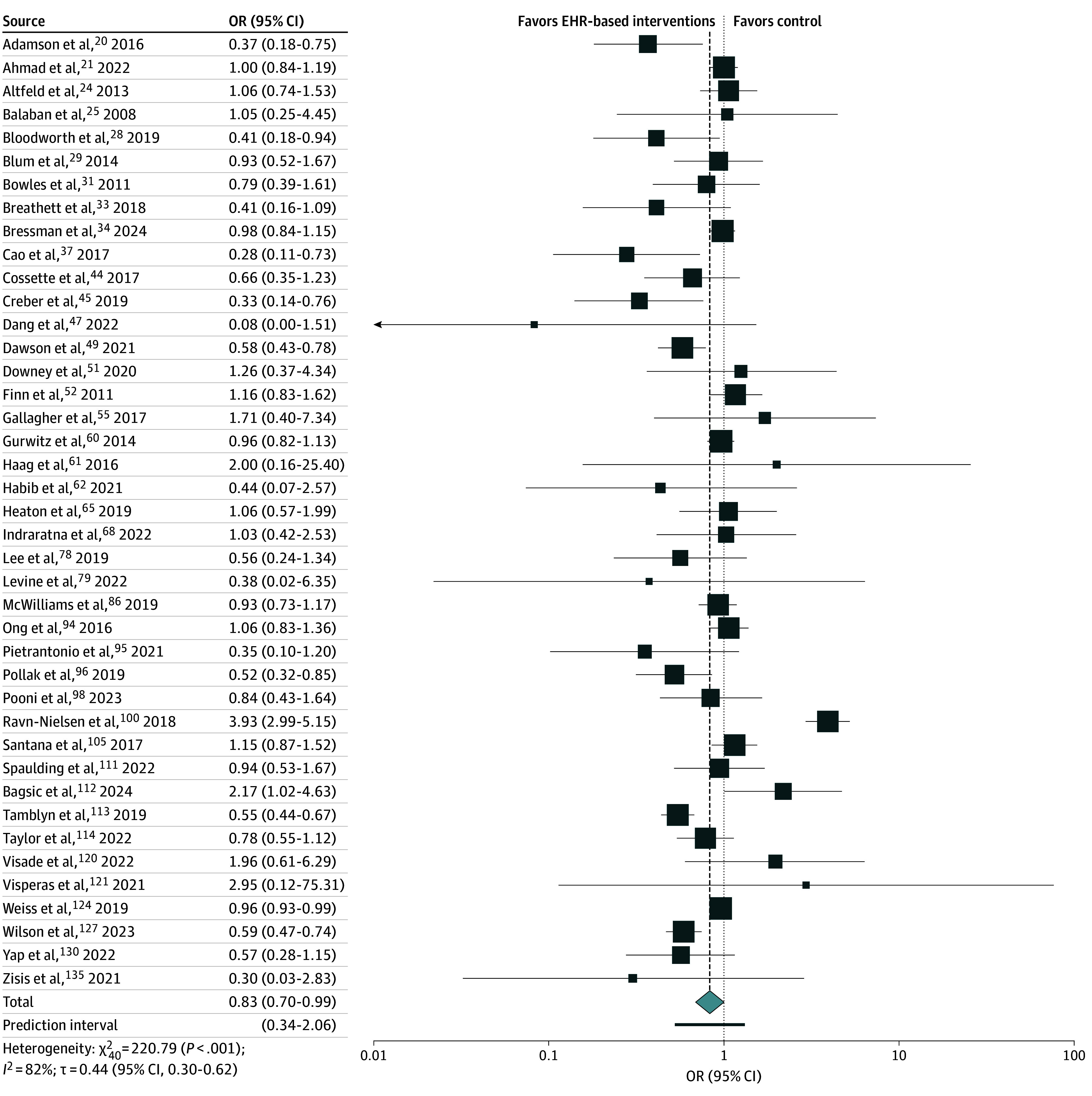
Meta-Analysis Results for Odds Ratios (ORs) of 30-Day All Cause Readmissions Comparing Intervention and Control Arms Data are for 181 392 study participants. Size of boxes represents study weight.

Of the 116 included RCTs, 41 (35%) including 181 392 participants reported data on 30-day all-cause readmission.^20,21,24,25,28,29,31,33,34,37,44,45,47,49,51,52,55,60,61,62,65,68,78,79,86,94,95,96,98,100,105,111,112,113,114,120,121,124,127,130,135^ Compared with control arms, the use of EHR-based interventions was associated with a significant reduction in the odds of 30-day all-cause readmission (pooled OR, 0.83; 95% CI, 0.70-0.99; PI, 0.34-2.06) (Figure 2). Significant heterogeneity was also found (*I*^2^ = 82%; τ = 0.44 [95% CI, 0.30-0.62]; *P* < .001). Results of the subgroup analyses and univariable meta-regression to explore heterogeneity are presented in Table 2. When investigating differences in study populations by proportion of males below and above the mean (53%) included across studies, meta-regression analysis found sex to significantly modify the effect size across studies (explained *R*^2^ = 11%; *P* = .02). EHR interventions in studies including populations with disproportionately more males were also associated with a significant reduction in the odds of readmission (OR, 0.66; 95% CI, 0.52-0.84), but there was no association in studies with fewer males (OR, 0.97; 95% CI, 0.76-1.25). Furthermore, reductions in readmissions were noted for studies in which the mean age was greater than 67 years (OR, 0.68; 95% CI, 0.52-0.89), the intervention was not entirely supported by health care workers (OR, 0.78; 95% CI, 0.62-0.99), and the intervention included fewer than 3 components (OR, 0.72; 95% CI, 0.58-0.89); all were associated with a significant decrease in the odds of readmission, but meta-regression analyses did not identify statistically significant differences.

**Table 2.** Summary of Subgroup and Meta-Regression Analyses for Pooled Odds Ratios of 30-Day All-Cause Readmissions

Characteristic	Studies, No. (%) (n = 41)			Odds ratio (95% CI)			Explained *R*^2^^a^	*P* value^a^
	Yes	No	Unknown or NS	Yes	No	Unknown or NS		
Proportion of males >53%^b^	19 (46)	21 (56)	1 (3)	0.66 (0.52-0.84)	0.97 (0.76-1.25)	1.06 (0.74-1.53)	11	.02
Age >67 y	12 (29)	17 (42)	12 (29)	0.68 (0.52-0.89)	0.89 (0.72-1.1)	1.01 (0.65-1.57)	15	.19
Publication year >2019	18 (44)	23 (56)	NA	0.82 (0.66-1.01)	0.83 (0.64-1.07)	NA	0	.91
>204 Participants	26 (63)	15 (37)	NA	0.84 (0.68-1.03)	0.81 (0.54-1.23)	NA	0	.90
Admitted to cardiology	9 (22)	32 (78)	NA	0.77 (0.52-1.14)	0.85 (0.69-1.05)	NA	0	.54
HF admission diagnosis	10 (24)	31 (76)	NA	0.84 (0.62-1.13)	0.84 (0.67-1.05)	NA	0	.77
Intervention was entirely supported by health care professionals	23 (56)	18 (44)	NA	0.88 (0.68-1.14)	0.78 (0.62-0.99)	NA	0	.39
Used Epic Systems EHR	8 (20)	33 (80)	NA	0.97 (0.86-1.08)	0.81 (0.65-1.02)	NA	0	.75
Case management	18 (44)	23 (56)	NA	0.92 (0.70-1.21)	0.78 (0.62-0.98)	NA	0	.29
Telemonitoring	18 (44)	23 (56)	NA	0.81 (0.64-1.03)	0.84 (0.65-1.08)	NA	0	.77
Patient education	12 (29)	29 (71)	NA	0.82 (0.57-1.18)	0.83 (0.67-1.03)	NA	0	.93
Medication reconciliation	13 (32)	28 (68)	NA	0.90 (0.57-1.44)	0.83 (0.72-0.96)	NA	0	.43
≥2 Intervention components	35 (85)	6 (15)	NA	0.86 (0.70-1.04)	0.73 (0.44-1.20)	NA	0	.46
≥3 Intervention components	25 (61)	16 (39)	NA	0.95 (0.73-1.23)	0.72 (0.58-0.89)	NA	8	.07

Abbreviations: EHR, electronic health record; HF, heart failure, NA, not applicable; NS, not specified.

^a^ *P* value and *R*^2^ are results of meta-regression analyses.

^b^ Does not equal mean percentage of males in Table 1 as this is specific to studies reporting 30-day all cause readmissions.

Pooled results of additional 30-day readmission outcomes, including outcomes at 90 days and at 6, 12, and 24 months, can be found in Table 3 and eFigures 1 to 5 in Supplement 1. Among these outcomes, EHR-based interventions were associated with significantly reduced odds of 90-day all-cause readmission (pooled OR, 0.72 [95% CI, 0.54-0.96]; PI, 0.33-1.55; *I*^2^ = 78%; τ = 0.34 [95% CI, 0.19-1.00]).

**Table 3.** Results for Meta-Analysis for Additional Outcomes

Outcome	Studies, No.	Participants, No.	Pooled OR (95% CI)	*I*^2^, %	*P* value
**30 d**					
Unplanned readmission	6	1262	0.84 (0.51-1.40)	36	.17
Composite outcome	4	4907	0.83 (0.55-1.26)	69	.02
**90 d**					
All-cause readmission	15	13 281	0.72 (0.54-0.96)	78	<.001
Unplanned readmission	3	461	0.74 (0.24-2.25)	42	.18
Composite outcome^a^	NA	NA	NA	NA	NA
**6 mo**					
All-cause readmission	16	8466	0.83 (0.49-1.42)	96	<.001
Unplanned readmission^a^	NA	NA	NA	NA	NA
Composite outcome	5	3783	1.02 (0.96-1.09)	0	.97
**12 mo**					
All-cause readmission	8	2147	0.46 (0.20-1.05)	81	<.001
Unplanned readmission^a^	NA	NA	NA	NA	NA
Composite outcome^a^	NA	NA	NA	NA	NA
**24 mo**					
All-cause readmission	3	495	0.68 (0.23-2.01)	27	.25
Unplanned readmission^a^	NA	NA	NA	NA	NA
Composite outcome^a^	NA	NA	NA	NA	NA

Abbreviations: NA, not applicable; OR, odds ratio.

^a^ Outcome was reported in 2 or fewer studies, resulting in insufficient data for a meta-analysis. Composite outcome includes the end point of all-cause readmission and death.

### Risk of Bias

When risk of bias was assessed, 9 studies (8%) were assessed as having high risk^21,25,36,60,63,65,76,77,135^ (eTable 5 and eFigure 6 in Supplement 1). Reasons for high risk of bias included deviation from the intended intervention (5 [56%]),^36,60,63,65,135^ measurement of the outcome (2 [22%]),^21,25^ and selection of the reported results (2 [22%]).^76,77^

There was no visual evidence of publication bias among studies reporting the primary outcome (eFigure 7 in Supplement 1). There was also no statistical evidence of risk of publication bias based on the Egger test.

## Discussion

In this systematic review and meta-analysis, we summarized the current evidence on the efficacy of EHR-delivered interventions in reducing the risk of 30-day all-cause hospital readmission. We found that EHR-based interventions were associated with a reduction in the risk of 30-day and 90-day all-cause readmission by 17% and 28%, respectively, and intervention performance was better in study populations composed mostly of males and older participants, when participants were at least partially supported by health care professionals, and when the intervention included fewer than 3 components. Taken together, these results suggest that EHR-based interventions may be associated with reductions in hospital readmissions, with significant heterogeneity in the overall effect estimate that was partially explained by key study characteristics.

Our findings are similar to those demonstrated in Leppin and colleagues’^11^ 2014 systematic review and meta-analysis of existing evidence from 42 RCTs aimed at preventing 30-day hospital readmissions. However, in this prior review, interventions were not limited to those integrated into an EHR and were diverse. Case management, patient education, home visits, and self-management support were the most common intervention components. In contrast, the most common intervention components in the present study were telemonitoring, case management, telephone follow-up, and medication reconciliation. Furthermore, similar to our study, most of the populations in the study by Leppin et al^11^ included people with heart failure, in whom an 18% reduction in 30-day all-cause readmission was found. Additional systematic reviews have also assessed interventions to reduce hospital readmissions, generally finding improvements in outcomes.^136,137,138,139^ However, most could not perform a meta-analysis and did not require interventions to be integrated into an EHR. Outcomes beyond 30 days were also not investigated, and as our study suggests, EHR-based interventions may also be associated with reductions in longer-term readmission outcomes.

This study provides evidence for greater integration of interventions into EHRs to reduce hospital readmissions, which is promising given the uptake of EHRs in modern health care. A recent study aimed to investigate adoption of EHR-based systems across the US using data collected from the American Hospital Association.^140^ From 2009 to 2019, adoption rates for basic EHR systems steadily rose from 6.6% to 81.2%, while comprehensive EHR system adoption increased from 3.6% to 63.2% over the same time frame. Moreover, 82% and 87% of hospitals with a basic and comprehensive EHR system, respectively, were reported to offer telemedicine. This suggests that the availability and infrastructure required for EHR-based interventions is widespread. EHRs have several benefits, including improved clinical decision-making, triage decisions, and collaboration as well as automation of tasks, as previously highlighted.^141^ However, barriers to the acceptance of EHRs among physicians and nurses exist. A recent scoping review^142^ of 21 studies investigated obstacles to the implementation of EHRs; these included limited training to use complex software, high financial expenditures, time-related barriers, and social barriers, such as interference with the physician-patient relationship. Although EHRs are widespread, reducing barriers for meaningful use is essential to enable effective interventions beyond research settings.

Our subgroup analyses identified several findings of note. We found interventions at least partially supported by a health care professional and those designed with fewer than 3 components to be associated with a significant reduction in hospital readmissions. Patient engagement has been previously shown to be associated with improved health outcomes, adherence, and self-efficiency,^143^ and more specifically, access to EHRs among patients with diabetes has been shown to be associated with improved patient safety,^144^ highlighting the potential value of partially supported EHR-based interventions in which patients and/or caregivers are actively involved. Our findings also suggest that less complex and streamlined interventions may be optimal for reducing readmissions, although the literature remains inconsistent, with both multicomponent^11^ and single-component^145^ interventions showing benefits as well as no difference.^139^ Moreover, EHR-based interventions were associated with a reduction in the risk of readmission in older study populations, highlighting their potential importance for this group, which experiences the highest rates of readmission.^146^ In addition, interventions tested in populations with a disproportionately higher number of males were associated with greater reductions in readmissions than those tested in populations with a greater proportion of females. Previous research has shown that there is an underrepresentation of females in RCTs,^147,148,149^ including in trial planning,^150^ which may result in trial interventions that are not adequately designed or tailored to the specific needs of females. As such, our results may demonstrate a potential need for sex- and gender-related considerations in the development of interventions to reduce readmissions. Our subgroup analyses, while potentially informative for the design of future EHR-based interventions, should be interpreted with caution given the possibility of selection bias and the exploratory nature of subgroup analyses; they suggest a need for further investigation.

Most of the included studies were conducted in the US, with one of the lowest numbers of hospital beds per capita among Organisation for Economic Co-operation and Development countries, at 2.8 beds per 1000 population.^151^ Due to the high demand, hospital stays in the US are also among the shortest,^151^ often necessitating early or precarious discharge decisions for patients, with readmission as a potential consequence.^152,153^ Despite the longstanding national priority to reduce 30-day readmissions,^9^ a report from the Agency for Healthcare Research and Quality^4^ revealed 30-day all-cause readmission rates to have remained stable at 13.9 per 100 index admissions from 2016 to 2020. Moreover, considering that the initial reductions in readmissions observed in the US under the Hospital Readmission Reduction Program may have resulted from hospitals increasing the use of observation stays,^154^ along with the program’s inequitable effects,^155^ there is still a need to develop strategies and interventions to reduce hospital readmissions. Our study suggests a potential solution to this problem, and further exploration on how EHR interventions can be developed and implemented across the US and other jurisdictions is warranted.

### Limitations

This study has limitations. Most of the studies were conducted in the US, and their populations predominantly included individuals with a heart failure admission diagnosis with a cardiology attending service, which may limit the generalizability of our results. However, this reflects the nature of published literature and highlights the need to further investigate the impacts of EHR-based interventions on readmissions in diverse health systems and populations. Moreover, although we ensured that interventions were embedded into EHRs in some capacity, many studies did not report specifically how the intervention was integrated or the type of EHR system used. The definition of an EHR for our review was also based on the World Health Organization criteria,^14^ which were purposively chosen as they were globally accepted; this may have allowed for a broader range of studies, as study inclusion and exclusion were based on authors reporting that the intervention arm was EHR based and the control arm was not. We also noted that terminology used to report readmission outcomes varied between studies; for example, studies reported all-cause readmission data but listed them as readmission rates, which limited their inclusion in pooled meta-analyses. Any potential errors in extracting outcomes were mitigated through completing this process in duplicate. Together, these limitations highlight the need to improve reporting on how interventions are integrated into EHRs and use of standardized definitions for readmission outcomes.

## Conclusions

This systematic review and meta-analysis synthesized available evidence on EHR-based interventions designed to reduce the risk of hospital readmissions compared with control arms. We found that the use of EHR-based interventions was associated with a reduction in 30-day and 90-day hospital readmissions. Our findings support further efforts to implement and evaluate EHR-based solutions to address this global health system challenge in the rapidly evolving era of information technology. Future research should focus on understanding which additional components of EHR interventions drive their effectiveness and for whom. These insights can inform the design of more effective, patient-centered interventions that leverage EHR capabilities to improve care transitions and reduce preventable hospital readmissions.

## Notes

Supplement 1. **eTable 1.** Database-Specific Search Strategies **eTable 2.** Definitions of Variables Used to Categorize EHR-Based interventions **eTable 3.** Summary of Intervention Types Among Studies With Multicomponent Interventions **eTable 4.** Study and Participant Characteristics **eFigure 1.** Summary of Meta-Analysis for Odds Ratios of 30-Day Secondary Outcomes **eFigure 2.** Summary of Meta-Analysis for Odds Ratios of 90-Day Secondary Outcomes **eFigure 3.** Summary of Meta-Analysis for Odds Ratios of 6-Month Secondary Outcomes **eFigure 4.** Summary of Meta-Analysis for Odds Ratios of 12-Month All-Cause Readmissions **eFigure 5.** Summary of Meta-Analysis for Odds Ratios of 24-Month All-Cause Readmissions **eTable 5.** Revised Cochrane Risk-of-Bias Tool for Randomized Controlled Trials, Excluding Conference Abstracts **eFigure 6.** Assessment of Quality of Included Studies, Excluding Conference Abstracts, Using the Revised Cochrane Risk-of-Bias Tool for Randomized Clinical Trials **eFigure 7.** Funnel Plot of Publication Bias **eAppendix.** Reference List of Included Studies

Supplement 2.
Data Sharing Statement
