# Supplementary material for: Electronic Health Record Interventions to Reduce Risk of Hospital Readmissions: A Systematic Review and Meta-Analysis
Source: JAMA Netw Open. 2025 Jul 17;8(7):e2521785. doi: 10.1001/jamanetworkopen.2025.21785 (PMC12272288; doi:10.1001/jamanetworkopen.2025.21785)
Supplement: Supplement 1. — eTable 1. Database-Specific Search Strategies eTable 2. Definitions of Variables Used to Categorize EHR-Based interventions eTable 3. Summary of Intervention Types Among Studies With Multicomponent Interventions eTable 4. Study and Participant Characteristics eFigure 1. Summary of Meta-Analysis for Odds Ratios of 30-Day Secondary Outcomes eFigure 2. Summary of Meta-Analysis for Odds Ratios of 90-Day Secondary Outcomes eFigure 3. Summary of Meta-Analysis for Odds Ratios of 6-Month Secondary Outcomes eFigure 4. Summary of Meta-Analysis for Odds Ratios of 12-Month All-Cause Readmissions eFigure 5. Summary of Meta-Analysis for Odds Ratios of 24-Month All-Cause Readmissions eTable 5. Revised Cochrane Risk-of-Bias Tool for Randomized Controlled Trials, Excluding Conference Abstracts eFigure 6. Assessment of Quality of Included Studies, Excluding Conference Abstracts, Using the Revised Cochrane Risk-of-Bias Tool for Randomized Clinical Trials eFigure 7. Funnel Plot of Publication Bias eAppendix. Reference List of Included Studies [file jamanetwopen-e2521785-s001.pdf]

Supplementary Online Content

Pattar BSB, Ackroyd A, Sevinc E, et al. Electronic health record interventions to reduce risk of hospital readmissions: a systematic review and meta-analysis. *JAMA Netw Open*. 2025;8(7):e2521785. doi:10.1001/jamanetworkopen.2025.21785

eTable 1. Database-Specific Search Strategies
eTable 2. Definitions of Variables Used to Categorize EHR-Based interventions
eTable 3. Summary of Intervention Types Among Studies With Multicomponent Interventions
eTable 4. Study and Participant Characteristics
eFigure 1. Summary of Meta-Analysis for Odds Ratios of 30-Day Secondary Outcomes
eFigure 2. Summary of Meta-Analysis for Odds Ratios of 90-Day Secondary Outcomes
eFigure 3. Summary of Meta-Analysis for Odds Ratios of 6-Month Secondary Outcomes
eFigure 4. Summary of Meta-Analysis for Odds Ratios of 12-Month All-Cause Readmissions
eFigure 5. Summary of Meta-Analysis for Odds Ratios of 24-Month All-Cause Readmissions
eTable 5. Revised Cochrane Risk-of-Bias Tool for Randomized Controlled Trials, Excluding Conference Abstracts
eFigure 6. Assessment of Quality of Included Studies, Excluding Conference Abstracts, Using the Revised Cochrane Risk-of-Bias Tool for Randomized Clinical Trials
eFigure 7. Funnel Plot of Publication Bias
eAppendix. Reference List of Included Studies

This supplementary material has been provided by the authors to give readers additional information about their work.

© 2025 Pattar BSB et al. *JAMA Network Open*.

eTable 1. Database-Specific Search Strategies.

**MEDLINE**	
1	exp Patient Discharge/
2	(patient adj2 discharg*).tw,kf.
3	(hospital adj2 discharg*).tw,kf.
4	exp Patient Care Management/
5	(patient adj2 manag*).tw,kf.
6	(dischar* adj2 plan*).tw,kf.
7	exp "Continuity of Patient Care"/
8	exp Patient Education as Topic/
9	(patient adj3 educat*).tw,kf
10	exp Patient Care Planning/
11	exp Managed Care Programs/
12	(patient adj2 car*).tw,kf.
13	primary care.tw,kf.
14	((discharg* or care) adj2 bundle).tw,kf
15	exp Risk Reduction Behavior/
16	(discharge adj2 instruct*).tw,kf.
17	individual* plan*.tw,kf.
18	exp Medication Adherence/
19	(medication adj2 adher*).tw,kf.
20	follow-up.tw,kf.
21	care plan*.tw,kf.
22	care pathway*.tw,kf
23	exp Medication Reconciliation/
24	exp "Continuity of Patient Care"/
25	transition.tw,kf.
26	enhanced recovery after surger*.tw,kf.
27	ERAS.tw,kf.
28	exp Decision Support Systems, Clinical/
29	postdischarg*.tw,kf.
30	post discharg*.tw,kf.
31	1 or 2 or 3 or 4 or 5 or 6 or 7 or 8 or 9 or 10 or 11 or 12 or 13 or 14 or 15 or 16 or 17 or 18 or 19 or 20 or 21 or 22 or 23 or 24 or 25 or 26 or 27 or 28 or 29 or 30
32	readmission.tw,kf.
33	rehospital*.tw,kf.
34	return visit*.tw,kf
35	(return adj2 emergenc*).tw,kf
36	(return adj2 hospital).tw,kf
37	(return adj2 urgent care).tw,kf.
38	(readmi* adj2 emergenc*).tw,kf
39	(readmi* adj2 hospital).tw,kf
40	(readmi* adj2 urgent care).tw,kf

© 2025 Pattar BSB et al. *JAMA Network Open*.

41	(readmi* adj2 after hours).tw,kf.
42	exp Patient Readmission/
43	readmit*.tw,kf
44	32 or 33 or 34 or 35 or 36 or 37 or 38 or 39 or 40 or 41 or 42 or 43
45	31 and 44
46	(Randomized Controlled Trial or Controlled Clinical Trial or Pragmatic Clinical Trial or Equivalence Trial or Clinical Trial, Phase III).pt.
47	Randomized Controlled Trial/
48	exp Randomized Controlled Trials as Topic/
49	Controlled Clinical Trial/
50	exp Controlled Clinical Trials as Topic/
51	Randomization/
52	Random Allocation/
53	Double-Blind Method/
54	Single-Blind Method/
55	Single-Blind Studies/
56	Placebos/
57	Control Groups/
58	(random* or sham or placebo*).ti,ab,hw,kf.
59	((singl* or doubl*) adj (blind* or dumm* or mask*)).ti,ab,hw,kf.
60	((tripl* or trebl*) adj (blind* or dumm* or mask*)).ti,ab,hw,kf.
61	(control* adj3 (study or studies or trial* or group*)).ti,ab,kf.
62	(Nonrandom* or non random* or non-random* or quasi-random* or quasirandom*).ti,ab,hw,kf.
63	allocated.ti,ab,hw
64	((open label or open-label) adj5 (study or studies or trial*)).ti,ab,hw,kf.
65	((equivalence or superiority or non-inferiority or noninferiority) adj3 (study or studies or trial*)).ti,ab,hw,kf.
66	(pragmatic study or pragmatic studies).ti,ab,hw,kf.
67	((pragmatic or practical) adj3 trial*).ti,ab,hw,kf.
68	((quasiexperimental or quasi-experimental) adj3 (study or studies or trial*)).ti,ab,hw,kf.
69	(phase adj3 (III or "3") adj3 (study or studies or trial*)).ti,hw,kf
70	exp Child/
71	exp Pediatrics/
72	exp Infant, Newborn/
73	child*.tw,kf.
74	p?ediatric*.tw,kf.
75	newborn*.tw,kf
76	neonat*.tw,kf.
77	exp Adolescent/
78	adolescen*.tw,kf.
79	youth*.tw,kf
80	teen*.tw,kf.
81	infant*.tw,kf.

© 2025 Pattar BSB et al. *JAMA Network Open*.

82	70 or 71 or 72 or 73 or 74 or 75 or 76 or 77 or 78 or 79 or 80 or 81
83	46 or 47 or 48 or 49 or 50 or 51 or 52 or 53 or 54 or 55 or 56 or 57 or 58 or 59 or 60 or 61 or 62 or 63 or 64 or 65 or 66 or 67 or 68 or 69
84	45 and 83
85	84 not 82
86	exp Electronic Health Records/
87	electronic health record*.tw,kf.
88	EHR.tw,kf
89	86 or 87 or 88
90	31 and 44 and 83 and 89
91	90 NOT 82
**Embase**	
1	exp hospital discharge/
2	(patient adj2 discharg*).tw,kf.
3	(hospital adj2 discharg*).tw,kf.
4	exp patient care/
5	(patient adj2 manag*).tw,kf.
6	(dischar* adj2 plan*).tw,kf.
7	(patient adj3 educat*).tw,kf
8	exp patient care planning/
9	managed care program*.tw,kf.
10	(patient adj2 car*).tw,kf.
11	primary care.tw,kf.
12	((discharg* or care) adj2 bundle).tw,kf.
13	exp risk reduction/
14	(discharge adj2 instruct*).tw,kf.
15	individual* plan*.tw,kf.
16	exp medication compliance/
17	(medication adj2 adher*).tw,kf.
18	care plan*.tw,kf.
19	care pathway*.tw,kf.
20	exp medication therapy management/
21	transition.tw,kf.
22	enhanced recovery after surger*.tw,kf.
23	ERAS.tw,kf.
24	exp clinical decision support system/
25	postdischarg*.tw,kf.
26	post discharg*.tw,kf.
27	exp hospital readmission/
28	readmission.tw,kf.
29	rehospital*.tw,kf.
30	return visit*.tw,kf.
31	(return adj2 emergenc*).tw,kf.
32	(return adj2 hospital).tw,kf
33	(return adj2 urgent care).tw,kf.

© 2025 Pattar BSB et al. *JAMA Network Open*.

34	(readmi* adj2 emergenc*).tw,kf.
35	(readmi* adj2 hospital).tw,kf.
36	(readmi* adj2 urgent care).tw,kf.
37	(readmi* adj2 after hours).tw,kf.
38	readmit*.tw,kf.
39	exp randomized controlled trial/
40	exp Randomized Controlled Trials as Topic/
41	exp randomization/
42	random allocation.tw,kf.
43	exp double blind procedure/
44	exp single blind procedure/
45	single blind stud*.tw,kf.
46	exp placebo/
47	exp control group/
48	(random* or sham or placebo*).ti,ab,hw,kf.
49	((singl* or doubl*) adj (blind* or dumm* or mask*)).ti,ab,hw,kf
50	((tripl* or trebl*) adj (blind* or dumm* or mask*)).ti,ab,hw,kf.
51	(control* adj3 (study or studies or trial* or group*)).ti,ab,kf.
52	(Nonrandom* or non random* or non-random* or quasi-random* or quasirandom*).ti,ab,hw,kf.
53	allocated.ti,ab,hw.
54	((open label or open-label) adj5 (study or studies or trial*)).ti,ab,hw,kf.
55	((equivalence or superiority or non-inferiority or noninferiority) adj3 (study or studies or trial*)).ti,ab,hw,kf.
56	(pragmatic study or pragmatic studies).ti,ab,hw,kf.
57	((pragmatic or practical) adj3 trial*).ti,ab,hw,kf.
58	((quasiexperimental or quasi-experimental) adj3 (study or studies or trial*)).ti,ab,hw,kf.
59	(phase adj3 (III or "3") adj3 (study or studies or trial*)).ti,hw,kf.
60	(Randomized Controlled Trial or Controlled Clinical Trial or Pragmatic Clinical Trial or Equivalence Trial or Clinical Trial, Phase III).ti,ab,hw,kf.
61	39 or 40 or 41 or 42 or 43 or 44 or 45 or 46 or 47 or 48 or 49 or 50 or 51 or 52 or 53 or 54 or 55 or 56 or 57 or 58 or 59 or 60
62	exp child/
63	exp pediatrics/
64	exp infant/
65	child*.tw,kf.
66	p?ediatric*.tw,kf.
67	newborn*.tw,kf.
68	neonat*.tw,kf.
69	exp adolescent/
70	adolescen*.tw,kf.
71	exp juvenile/
72	youth*.tw,kf.
73	teen*.tw,kf.

© 2025 Pattar BSB et al. *JAMA Network Open*.

74	infant*.tw,kf.
75	62 or 63 or 64 or 65 or 66 or 67 or 68 or 69 or 70 or 71 or 72 or 73 or 74
76	1 or 2 or 3 or 4 or 5 or 6 or 7 or 8 or 9 or 10 or 11 or 12 or 13 or 14 or 15 or 16 or 17 or 18 or 19 or 20 or 21 or 22 or 23 or 24 or 25 or 26
77	27 or 28 or 29 or 30 or 31 or 32 or 33 or 34 or 35 or 36 or 37 or 38
78	61 and 76 and 77
79	78 not 75
80	exp electronic health record/
81	electronic health record*.tw,kf.
82	EHR.tw,kf.
83	61 and 76 and 77 and 83
84	84 not 75
**Cochrane Central Register of Controlled Trials**	
[Title Abstract Keyword] "hospital discharge" OR "discharge plan" OR "medication compliance" OR "care pathway" OR "patient care" OR ERAS<br>AND<br>[Record Title] readmi* OR re-admi* OR rehospital* OR re-hospital* OR return* OR visit*	
**CINAHL Plus with Full Text**	
"hospital discharge" OR "discharge plan" OR "medication compliance" OR "care pathway" OR "patient care" OR ERAS OR post-discharg* [abstract]<br>AND<br>Readmi* OR rehospital* [abstract]<br>NOT<br>systematic reviews or meta analysis or meta- analysis [abstract]	
**Clinicaltrials.gov**	
Expert Search Query: ("hospital discharge" OR "discharge plan" OR "medication compliance" OR "care pathway" OR "patient care" OR ERAS) AND (readmi* OR re-admi* OR rehospital* OR re-hospital* OR return*) AND (EHR OR EMR OR "Electronic Health Records" OR "electronic medical records")	

© 2025 Pattar BSB et al. *JAMA Network Open*.

eTable 2. Definitions of variables used to categorize EHR-based interventions.

Variable	Definition
**Intervention Component**	
Case Management	Coordinating care or resources without focusing on self-management, more extensively than in the control arm.
Clinician Continuity	Stronger provider involvement across inpatient and outpatient care, beyond what control arm had.
Discharge Planning	Introducing a structured approach to discharge planning that was not present in the control arm.
Follow-Up Scheduled	Arranging follow-up before discharge, not consistently done in control arm.
Home Visits	In-person visits to the patient's home by the care team, not part of the control arm.
Making Requisites	Better use or quality of current services compared to use in the control arm.
Medication Reconciliation	Reconciliation or tailored education to support medication use, often pharmacist-led but not necessarily.
Patient-Centered Discharge Instructions	Improved discharge materials made more usable or personalized versus control arm.
Patient Education	Education on diagnosis or treatment (not self-management), which is not given in the control arm.
Patient Hotline	Accessible patient-initiated contact line, more available or functional than in the control arm.
Rehabilitation Intervention	Rehabilitation activities to improve function, not diagnosis-specific, and not part of the control arm.
Self-Management	Education or coaching aimed at enhancing self-care skills, which is absent in the control arm.
Streamlining	More efficient or clearly assigned service delivery, which is lacking in the control arm.
Telemonitoring	Use of remote tools for patients to send health data, possibly with symptom tracking, which not used in the control arm.
Telephone Follow-Up	Provider-initiated calls or video check-ins after discharge, absent in the control arm.
Timely Follow-Up	Faster post-discharge contact that is delayed or missing in the control arm.
Timely PCP Communication	Quicker updates to primary care providers than what occurs in the control arm.
Other	Unique components such as caregiver support or peer mentoring not in the control arm.
**Intervention System**	
Computer	A software system used to manage and monitor participant's uploaded data.
EHR	A real-time health data record, accessible in immediate and secured fashion to authorized users.
Electronic Messaging	Communication with participants via email or a short message service.

© 2025 Pattar BSB et al. *JAMA Network Open*.

Internet or Website	A website accessible through the internet where participants had access to educational materials and could record symptoms.
App	An app downloaded to mobile devices where participants had access to educational materials and could record symptoms.
Robot	Supported participants by monitoring health status and symptoms, providing medication and exercise reminders, offering education, and displaying health trends over time.
Telehealth	Use of remote tools for patients to send health data, possibly with symptom tracking.
Other	Computer-controlled machine that stores, dispenses, and tracks data on medications.
**Function of EHR-Embedded Component**	
Communication with Health Care Provider	Exchange of health information between patients and providers, including sending health data, receiving feedback or education, and adjusting treatment plans based on reported information.
Communication with Peers	Participants could designate a support partner to receive nonadherence alerts, helping encourage adherence through direct support or accountability.
Other Psychotherapy	Software included monthly anxiety and depression assessments that were reviewed, allowing for adjustment in treatment or recommend psychotherapy follow-up.
Prompts and Alerts	Notified the healthcare team of abnormal data and offered decision support to guide appropriate clinical action.
Provider Monitoring	Reviewing patient data to assess progress, identify issues, and make informed decisions regarding treatment adjustments.
Self-Monitoring	Patients upload their health data and/or symptoms to a secure system for potential review by healthcare providers.
Screening	Participants upload their data for providers to review and determine if further testing was needed to confirm reported symptoms.
Transmission of Information	One-way communication between participants and the healthcare provider team.
Other	Discharge forms were completed and uploaded to the EHR, ensuring all care team members had access to the information.
**Intervention timing**	
Asynchronous	Exchange of information that does not occur in real time, such as secure messages, emails, or data reviewed by healthcare providers at a later time.
Synchronous	Real-time transfer and monitoring of data, where information is transmitted and evaluated immediately, which may trigger alerts or actions.
**Intervention Facilitation**	
Entirely Supported by Healthcare Provider	Fully delivered and managed by healthcare providers, with no active involvement from the patient or caregiver.
Partially Supported by Healthcare Provider	Involves both healthcare providers and patients or caregivers, requiring shared participation.

© 2025 Pattar BSB et al. *JAMA Network Open*.

Self-Administered	Primarily carried out by the patient or caregiver with minimal provider involvement.

Notes. Definition of intervention components adapted from Leppin et al. (2014; See citation #11). Definitions of system, function, timing, and facilitation adapted from Gagnon et. al (2022; See citation #14). Abbreviations: EHR: Electronic Health Record. PCP: Primary Care Provider.

© 2025 Pattar BSB et al. *JAMA Network Open*.

eTable 3. Summary of intervention types among studies with multicomponent interventions

	**n=91**
Case Management	45 (49)
Clinician Continuity	12 (13)
Discharge Planning	16 (18)
Follow-up Scheduled	6 (5)
Home Visits	4 (4)
Making Requisites	5 (5)
Medication Reconciliation	28 (31)
Patient-Centered Discharge Instructions	8 (9)
Patient Education	32 (35)
Patient Hotline	7 (8)
Rehabilitation Intervention	2 (2)
Self-Management	24 (26)
Streamlining	3 (3)
Telemonitoring	59 (65)
Telephone Follow-Up	35 (38)
Timely Follow-Up	10 (11)
Timely PCP Communication	5 (5)
Other	12 (13)

Notes. Multi-component intervention was defined as ≥2 intervention components. Results may not add up to 100% or n number of studies given multiple options being reported in a single study. Abbreviations: PCP: Primary Care Provider.

© 2025 Pattar BSB et al. *JAMA Network Open*.

eTable 4. Study and participant characteristics.

Author and Year	Patient Population and Setting	Admission Diagnosis and Service	Intervention	Control	Intervention Duration (years)	Follow-up time (years)	Number of participants recruited	EMR Type/Brand	Age	Female Sex (%)	Hypertension	Diabetes	Smoking	Outcomes
Adamson et al. 2016[1]	Patients who were Medicare-eligible; USA	HF; Cardiology	Uploaded pressures were made available to investigators from a wireless implantable hemodynamic monitoring system.	Standard of care HF management.	NR	1.5	245	CardioMicroelectromechanical system	C:71; I:70†	61.0%	NR	NR	C:39(35); I:36(33)	ACR: 30d
Ahmad et al. 2022[2]	Adults who had an NT-proBNP level of 500 pg/mL or more and received intravenous loop diuretics within 24 hours of admission; USA	HF; Cardiology	An alert displaying the predicted 1-year mortality rate, as well as other relevant information, was displayed to clinicians when they opened the order-entry portion of the medical record.	No alert displayed.	NR	1	3124	Epic Systems	66.6*	64.20%	NR	NR	NR	ACR: 30d
Anderson et al. 2023[3]	Patients above 40 years of age who were hospitalized for COPD; Denmark	COPD; NR	Telemonitoring intervention consisted of home measurement of oxygen saturation, heart rate, peak expiratory flow, body weight, and completion of a standardized questionnaire with yes or no questions.	Pharmacological and non-pharmacological treatment according to the clinical recommendations for COPD and regular follow-up at the respiratory outpatient clinic.	0.5	2	224	Tunstall Healthcare's Telemonitoring equipment and a certified server hosted by the Central Denmark Region accessible from the hospital	55.7*	46.1%	NR	NR	NR	ACR/CO: 6mo, 24mo
Asch et al. 2022[4]	Patients with preserved or reduced ejection fraction; USA	HF; Cardiology	Participants received a digital scale, electronic pill bottle, daily lottery incentives, and were monitored for weight changes. Verified results were sent to clinicians, with optional support partner alerts for nonadherence.	Received no further engagement with study personnel.	NR	1	566	NR	72.5*	29.0%	NR	C:44(43); I:45(43)	NR	CO: 12mo§
Altfeld et al. 2013[5]	Patients 65 and older lack post-discharge care support, have a high fall risk and in-depth psychosocial needs complicating care; USA	Non-specific; Geriatrics	A social work-based telephone intervention where referrals are generated through an automated daily report of hospital discharges utilizing risk criteria documented in the patient's EMR.	Conventional care given to all patients discharged from the medical center.	NR	0.08	906	NR	72.5*	65.40%	NR	NR	NR	ACR: 30d§
Balaban et al. 2008[6]	Admitted patients with an established PCP; USA	Admission to medical/surgical; Internal Medicine & Surgery	A user-friendly Patient Discharge Form is given to patients, electronically sent to RNs at their primary care site, followed by a call from a primary care RN, and reviewed by the PCP.	Discharge according to existing hospital practices and protocols.	NR	0.08	122	NR	67.3*	19.3%	C:34(40); I:74(44)	C:30(35); I:59(35)	NR	ACR: 30d
Benatar et al. 2003[7]	Heart failure; USA	HF; Cardiology	The home monitor transmits patient physiological data via a telephone line to a central server, accessible by caregivers online. Alarms for abnormal data are sent to an alphanumeric pager for timely response.	Home nurse visits included discussions on diet, symptom recognition, medication compliance, symptom and vital sign assessments, with physician notification if necessary.	0.25	1	216	AvidCare Corporation	C:62; I:62†	44.8%	NR	NR	NR	CSR: 90d, 6mo, 12mo (HF)
Bentley et al. 2014[8]	Previous admission in 12 months with COPD as primary or secondary documented reason for hospitalisation; England	COPD exacerbation; NR	The telehealth system allows patients to monitor daily vital signs. If signs or symptoms are abnormal or monitoring is missed, clinician alerts are triggered for timely intervention.	Home visit trial info pack and discharge home visit.	0.17	0.5	63	Doc@Home	59.6*	38.4%	NR	C:22(15); I:21(15)	NR	ACR/CO: 8mo
Bloodworth et al. 2019[9]	Adult patients admitted for inpatient treatment of one of the following: AMI, PNA, HF, or COPD; USA	Acute MI, Pneumonia, HF, or COPD; NR	The Pharmacist Transition Coordinator facilitated outpatient transitions by accessing EMR records, performing medication reconciliation, addressing barriers to medication access or	Typical inpatient medical and pharmacy services.	0.5	0.5	256	Epic Systems	66.5*	47.7%	NR	C:180(26); I:185(27)	NR	ACR/RR: 30d, 90d, 6mo

© 2025 Pattar BSB et al. *JAMA Network Open*.

			adherence, resolving issues, and ensuring patients had a primary care provider or medical home.											
Blum et al. 2014[10]	Medicare patient being discharged from heart failure service; USA	HF; Cardiology	Remote monitoring tracked daily weights, blood pressure, heart rate, and a 15-second heart rhythm strip. Data outside assigned parameters were flagged for a nurse practitioner, who contacted patients and adjusted medications, typically diuretics, as needed.	Written material about heart failure and self-management activities.	NR	4.42	206	Philips Electronics E-care System	63.5*	47.20%	NR	NR	NR	ACR/RR: 30d
Bonnet-Zamponi et al. 2013[11]	Individuals aged 70 and older without a poor chance of survival at 3 months; France	NR; Geriatrics	Discharge-planning intervention combining chronic drug review, education, and enhanced transition-of-care communication.	Usual Care.	NR	0.5	655	NR		55.2%	NR	C:177(56); I:172(54)	NR	RR: 6mo
Bowles et al. 2011[12]	Patients 55 and older discharged from hospital for HF as a primary or secondary diagnosis and referred to home care with the study agency; USA	HF; Cardiology	Received wireless telehealth equipment for daily monitoring. Nurses provided training, monitored data, conducted four video visits, and collaborated with home care nurses to address out-of-range readings, adjusting care or notifying physicians as needed.	At least five intermittent in-person skilled visits by a registered nurse over a 60-day episode to assess, teach, and case manage the patient's care.	0.12	0.5	218	Carematix, Inc	83.8*	57.7%	NR	NR	C:139(40); I:150(43)	ACR: 30d
Boxer et al. 2022[13]	Hospital discharge diagnosis for HF; USA	HF; Cardiology	Optimize medical therapy, patient education, and follow-up.	Usual Care.	0.16	0.16	671	NR	76*	35.5%	NR	NR	NR	Comp‡: 30d, 60d
Breathett et al. 2018[14]	Adult patients admitted with a primary diagnosis of HF; USA	HF; Cardiology	Education included one-on-one heart failure discussions. A tablet app provided personalized education on heart failure, nutrition, medication adherence, and lifestyle changes, while flagging patient questions for medical staff.	Education alone.	DOH	0.08	126	NR	60.5*	17.0%	C:(38); I:(63)	C:(24); I:(25)	C:(15); I:(9)	ACR: 30d
Bressman et al. 2024[15]	Primary care patients identified as medium to high risk; USA	NR; NR	Patients received automated check-in texts via Way To Health, asking if they needed help. Responses were routed to the EMR inbox for practice staff, with escalations handled during business hours.	Standard transitional care management telephone call from their practice within 2 business days of discharge	0.082	0.16	5051	Epic Systems	C:62; I:68†	25.0%	NR	NR	NR	ACR: 30d
Broadbent et al. 2018[16]	Patients with confirmed COPD, recent COPD-related admission, limited mobility, rural location, poor social support, aged 16-90, and FEV1/FVC <0.7 were recruited; New Zealand	COPD; Pulmonology	Intervention system monitored weekly health data, reminded patients about medication, encouraged rehabilitation exercises, provided COPD education, offered an "unwell" alert, and tracked health trends and inhaler use via Smartinhalers.	Patients received rehabilitation referrals, follow-up by their GP, respiratory clinic appointment, and Smartinhalers for adherence tracking, delivered a week post-discharge.	0.33	0.33	60	iRobi robot (Yujin Robot Limited, Korea)	57*	15.0%	C:52(23); I:44(19)	C:61(27); I:67(29)	NR	CSR: 4mo (Respiratory)
Cadman et al. 2017[17]	Adult patients Admitted with at least one prescribed medicine; UK	Non-specific (one prescribed medicine); Various	delivery of medicine reconciliation by a trained medication reconciliation pharmacist within 24 hours of admission and at the point of transfer of care out of hospital.	Usual care which may or may not consist of medication reconciliation	DOH	0.25	198	NR	59.1*	32.5%	C:93(66); I:84(61)	C:58(41); I:57(41)	C:88(62); I:86(62)	ACR: 90d
Cao et al. 2017[18]	Adult patients diagnosed with coronary heart disease discharged to home residence; Japan	Coronary heart disease diagnosis; Cardiology	During hospitalization, cardiologists and nurses collaborated on medication, treatment instructions, and discharge planning, followed by post discharge communication with community healthcare for continued patient care.	Usual Care.	NR	0.25	236	NR	66.2*	43.5%	NR	NR	NR	ACR: 30d, 90d
Casida et al. 2022[19]	Patients undergoing LVAD implantation for the first time; USA	HF; Cardiology	Usual care plus VAD Care App providing structured, comprehensive LVAD self-management content. Patients submit data, and abnormal results are flagged for RNs/NPs to intervene accordingly.	Usual care consisting of routine outpatient visits with the VAD care team.	0.5	0.5	40	NR	C:86; I:81†	24.3%	NR	NR	NR	UR: 30d, 90d, 6mo
Ceschi et al. 2021[20]	Patients aged 85 years or older, with more than 10 medications at hospital admission; Switzerland	Non-specific; NR	Medication reconciliation involved a pharmacy assistant creating a comprehensive medication list at admission. The clinical pharmacist reconciled discrepancies with the physician's list, proposed changes, and physicians finalized pharmacotherapy adjustments.	Standard, unstructured medication history process, without pharmacist consultation or access to EHR data from other hospitals, clinics, or pharmacies for reviewing medication lists and refill histories.	DOH	1.21	2198	NR	C:68.6; I:54.6†	38.80%	NR	NR	NR	ACR

© 2025 Pattar BSB et al. *JAMA Network Open*.

Chau et al. 2012[21]	Patients with moderate or severe COPD; Hong Kong	COPD; NR	Patients received a device kit monitored oxygen saturation, pulse rate, and respiration rate three times daily, transmitting data to an online platform.	Usual care, community nurse conducted home visits, providing education on self-care, symptom management, medication use, purse-lip breathing, lifestyle changes, and exercise, without additional interventions.	0.15	0.17	53	ASTRI telecare system	79*	52.5%	NR	C:708(40); I:690(37)	NR	ACR: 60d
Chaudhry et al. 2010[22]	Patients hospitalized with HF; USA	HF; Cardiology	Daily, toll-free calls, answering questions on health and HF symptoms using their phone keypad. Depression screening occurred every 30 days. Data were reviewed daily by coordinators on a secure internet site.	Usual care included educational materials and if needed, a scale.	0.49	0.49	1653	Tel-Assurance (Pharos Innovations)	61.7*	47.2%	NR	C:21(11); I:26(12)	NR	ACR/CO: 6mo
Cleland et al. 2005[23]	Patients with hospital admission associated with worsening heart failure; UK	HF; Cardiology	Usual care plus nurse telephone support and telemonitoring. Devices transmitted data to a hub, alerting study nurses to abnormal values for review and action, or to contact the patient's PCP.	Individualized written management plan that described what pharmacologic treatment they should receive. Patient management plan was sent to the patients PCP.	NR	0.66	253	NR	61.5*	21.0%	C:46(55); I:39(48)	C:22(27); I:21(26)	C:21(25); I:18(22)	ACR: 240d, 450d
Comin-Colet et al. 2016[24]	HF patients with either reduced ejection fraction or pre- served ejection fraction; Spain	HF; Cardiology	Multichannel service tracked biometric data and symptoms, managing alarms for out-of-range values, while household devices transmitted patient data to clinicians via Bluetooth and 3G technology.	Patients instructed to perform same determinations of bio-measures, record them and contact the nurse when these were out of range	0.5	0.5	188	Telefonica Soluciones S.A	57.9*	14.5%	NR	NR	NR	ACR: 6mo
Cossette et al. 2016[25]	Adults aged 65 or older who presented with at least one of the geriatric explicit criteria for potentially inappropriate medications; Canada	Mitral regurgitation; Geriatrics	A computerized alert system used EMR data to identify geriatric criteria without manual entry. The study pharmacist reviews and assesses the alerts daily for clinical relevance.	Usual care were not reviewed during the hospital stay. The study pharmacist assessed their clinical relevance after the patient was discharged.	DOH	0.08	321	Quadramed	72.8*	24.5%	C:101(81), I:93(78)	C:59(47); I:53(44)	NR	ACR: 30d
Creber et al. 2019[26]	Admitted adult patients who were English or Spanish-speaking; UK	Non-specific; NR	Access to portal which included care team info, medications, videos on medication use and side effects, MedlinePlus links, allergies, test orders/results, diet, vitals, pain reporting, communication with the care team, and rating functionality.	Usual care.	NR	0.08	316	Allscripts Sunrise	C:77; I:76.5†	50.7%	NR	C:670(44); I:694(44)	NR	ACR: 30d
Dabbs et al. 2016[27]	Adult patients that underwent transplantation; USA	Lung Transplant; NR	Usual care plus a smartphone app to record health data, track trends, receive alerts, and automatically upload critical indicators for research and coordinator notification.	Patients received scripted discharge instructions, a binder emphasizing daily self-management, adherence to the regimen, self-monitoring, and reporting abnormal health indicators to the transplant coordinator.	NR	1	201	Pocket PATH program	74.5*	0.0%	NR	NR	NR	ACR: 12mo
Dang et al. 2022[28]	Patients with IBD flare without surgical intervention; Netherlands	IBD; Gastroenterology or general internal medicine	Standardized follow-up by an IBD practice nurse and electronic health outcome monitoring through NoviSurvey.	Current approaches of hospital discharge alone.	NR	0.08	41	NoviSurvey	64.5*	47.5%	C:122(49); I:123(50)	C:133(53); I:112(45)	NR	ACR: 30d
Dar et al. 2009[29]	Adult patients with a primary diagnosis of HF and deemed fit for; England	HF; Cardiology	Telemonitoring equipment recorded vitals and symptoms daily. Data were reviewed by a nurse, triggering alerts for clinical deterioration, resulting in advice, medication changes, or care adjustments.	A home visit with self-monitoring advice, clinic reviews by a HF team, telephone support during working hours, lifestyle guidance, and medication optimization, with follow-up frequency decided by the team.	0.5	0.5	182	Honeywell HomMed	NR	59.40%	NR	C:18(37); I:12(26)	NR	ACR: 6mo
Dawson et al. 2021[30]	Adult patients who are at high risk of readmission; USA	Non-specific; NR	Standard care plus monitoring with equipment that recorded daily vital signs, transmitted to a cloud program. Alerts triggered when vitals were abnormal, prompting nurse follow-up and documentation in the EMR.	Standard care included teach-back education, medication reconciliation, and a follow-up phone call within 72 h of discharge.	0.08	0.08	1380	NR	63.1*	53.10%	C:96(89), I:108(100)	C:11(10); I:39(36)	NR	ACR/CO: 30d§

© 2025 Pattar BSB et al. *JAMA Network Open*.

Dendale et al. 2012[31]	Patients admitted with chronic HF and reduced ejection fraction; England	HF; Cardiology	Telemonitoring follow-up where patients recorded measurements which were forwarded to a central computer. When recordings were outside predefined limits, GP and HF clinic were alerted course of action was determined.	Standard HF care	0.5	0.5	160	NR	86.1*	66%	NR	NR	NR	ACR per patient
Downey et al. 2020[32]	Patients undergoing major elective abdominal surgery; England	Abdominal surgery; Surgery	Participants received monitoring equipment that transmitted data continuously to mobile device carried by nurses who reviewed alert when deviations from pre-set norms. Nurse made decision regarding response to alert.	Standard NEWS monitoring.	0.08	0.08	136	SensiumVitals® system	78.9*	58.0%	C:300(88); I:289(88)	C:151(44); I:127(39)	NR	ACR: 30d
Finn et al. 2011[33]	Admitted under the team attending(s); USA	Non-specific; NR	A NP was randomly assigned to 1 resident team to complete discharge paperwork, arrange follow-up appointments and prescriptions, communicate discharge plans with nursing and PCPs, and answer questions.	Scheduling of follow-up appointments was the responsibility of the team resident Nurses provided patient discharge education.	NR	0.08	999	NR	66.5*	53.0%	NR	NR	NR	ACR: 30d
Franchi et al. 2016[34]	Patients aged 75 years or over consecutively admitted to the participating wards; UK	Non-specific; Internal Medicine and geriatrics	Physicians in the intervention completed an e-learning program covering CGA concepts, geriatric pharmacology, prescription appropriateness, polypharmacy management, and tools for evaluating prescriptions and drug interactions.	E-learning program consisted only of a refresher on the basic notions of geriatric pharmacology using Module B as a weapon.	NR	1	697	INTERcheck® software	68.1*	26.3%	NR	NR	NR	RR: 12mo
Frederix et al. 2015[35]	Patients with an acute coronary syndrome; England	An acute coronary syndrome; Cardiology	Participants wore a motion sensor to track activity data, uploaded it to their online account, and received personalized automated feedback, encouraging gradual increases in physical activity.	Patients wore a modified motion sensor. The information was hidden from the patient with no feedback was delivered.	0.35	0.35	80	Yorbody company	52.1*	27%	NR	C:3(19); I:2(14)	NR	ACR: 125d
Gallagher et al. 2017[36]	Patients 21 years or older; spoke English or Spanish; and were discharged home on a loop diuretic; USA	HF; Cardiology	Provided with a pill bottle that transmitted data regarding the date and time it is opened. Data was monitored and participants were contacted when non adherent for two or more days per week.	Provided with a pill bottle that transmitted data regarding the date and time it is opened. Adherence data was recorded but not monitored.	0.08	0.08	40	GlowCap® system (Vitality, Inc., Los Angeles, CA)	C:86; I:86†	58.80%	NR	NR	NR	ACR: 30d
Giordano et al. 2009[37]	Patients hospitalised with confirmed diagnosis of HF and at least one hospitalisation in the previous year; Netherlands	HF; Cardiology	Home-based telemanagement involved multidisciplinary care via telephone, with ECG data transmitted to a workstation. Patients used portable devices, and nurses/doctors provided 24/7 support. Calls triggered data retrieval and symptom assessments.	All patients received HF education, covering daily weights, blood pressure monitoring, diet restrictions, and decompensation signs. UC patients were referred to primary care.	0.81	1	460	Card-Guard 2206	C:61; I:61†	42.0%	C:639(77); I:631(77)	C:378(46); I:394(48)	NR	ACR: 12mo
Goldberg et al. 2003[38]	Patients hospitalized with class III or IV heart failure; USA	HF; Cardiology	Intervention patients used the AlereNe system for daily weight and symptom tracking. Nurses monitored data and alerted physicians when readings exceeded pre-set ranges.	Instructed to contact their physician for significant weight increases or worsening heart failure symptoms. They brought weight logs to study visits, with follow-ups and calls at the physician's discretion.	0.46	0.5	280	AlereNet system (Alere Medical, Reno, Nev) - DayLink monitor	72.9*	2.5%	NR	NR	NR	ACR: 6mo
Goldman et al. 2014[39]	Hospitalized adults aged 55 years or older with community discharge; USA	Non-specific; Internal or family medicine, cardiology, or neurology department	Usual care plus RN disease education, AHCP booklet, and NP follow-up calls on days 1-3 and 6-10 for education, adherence checks, referrals, and PCP updates recorded in EMR.	All patients received usual discharge care, including RN instruction review. If requested, the pharmacy provided a 10-day medication supply, and a social worker assisted. The admitting team sent the discharge summary to the PCP.	0.03	0.5	700	NR	74.5*	40.9%	C:87(90); I:70(86)	C:49(50); I:37(46)	NR	ACR: 30d, 90d RR:6mo
Graumlich et al. 2009[40]	Inpatients discharged to home with high risk for readmission; USA	Non-specific; NR	Software facilitated discharge communication with patients, pharmacists, and physicians. Features included required fields, pick lists, alerts, reminders, and online references. It	Usual care included handwritten discharge forms on the day of discharge.	NR	0.5	631	NR	66.4*	59.2%	NR	NR	NR	ACR: 6mo

© 2025 Pattar BSB et al. *JAMA Network Open*.

			prompted test orders and generated discharge forms automatically.											
Gurwitz et al. 2014[41]	Patients aged 65 or older discharged from hospital to home; USA	Non-specific; NR	An automated system informed PCPs about patients discharged to home, detailing new medications, drug interaction warnings, dose change recommendations, and alerts for scheduling post-hospitalization visits within a week.	Usual approach to follow-up care at the time of hospital discharge	0.08	0.08	5077	Epic Systems	71.7*	36.0%	NR	NR	NR	ACR: 30d
Haag et al. 2016[42]	Elderly adults at high risk for an ED visit or readmission; USA	Non-specific; NR	MTM consultations post-discharge. Pharmacists reviewed EMR data for medication assessments, identified drug-related issues, and checked for prescribing omissions, then conducted phone consultations for medication optimization.	Usual care involved a pre-existing consultation without pharmacist intervention. Post-discharge, nurse practitioners visited patients within 3 days, reviewing medications and making changes, with follow-up calls as needed.	0.02	0.08	25	NR	80.2*	23.5%	C:33(62); I:28(53)	C:10(19); I:11(21)	C:53(100); I:53(100)	ACR: 30d
Habib et al. 2021[43]	Adult patients discharged home with medication prescriptions; USA	Non-specific; NR	Usual care plus training and access to the SAM mobile app, which matched prescribed and dispensed medications, generated patient-friendly lists, and offered features like pill images, adherence alerts, and drug information.	Usual care at discharge included medication reconciliation, written discharge prescription to be filled at their community pharmacy and potential instructions about their discharge prescription or medications changes.	0.08	0.08	66	NR	73.1*	49.8%	C:85(73); I:77(67)	C:35(30); I:26(23)	NR	ACR: 30d
Hajizadeh et al. 2020[44]	African American or Hispanic persons who were hospitalized for a COPD exacerbation; USA	COPD; Pulmonology	Pulmonary rehabilitation via telehealth at home or community centers over Zoom. A Nonin watch monitored vital signs, with a pulmonologist on-call during sessions, which included educational videos from SPR.	Standard, office-based pulmonary rehabilitation. Provided with an exercise peddler and a list of locations to encourage continuation of exercise and educational videos.	NR	0.5	266	NR	63*	44.5%	NR	NR	NR	CSR: 6mo (COPD)
Hale et al. 2016[45]	Patients who completed a HF telemonitoring program and had been hospitalized in the previous 24 months; Canada	HF; Cardiology	A remotely monitored electronic device that alerts participants when it is time to take their medications and a monitoring center that contacts participants and caregivers when medications are not taken.	Continued to use their usual medication reminder method.	0.25	0.25	29	MedSentry Medication Management System	77*	47%	NR	NR	NR	ACR: 90d
Heaton et al. 2019[46]	Patients discharged home with a primary diagnosis of acute MI, pneumonia, HF, COPD, or diabetes; USA	Acute MI, pneumonia, HF, COPD, or diabetes; NR	Hospital referrals with continuity of care documents to a call center. Student pharmacists scheduled appointments, while pharmacists performed medication reconciliation, reviews, counseling, and provided patient resources.	Usual care.	0.04	0.08	400	Epic Systems	63.6*	42.0%	NR	NR	NR	ACR: 30d
Ho et al. 2016[47]	Patients aged 20 years or older admitted with a diagnosis of COPD; Taiwan	COPD; Various	Telemonitoring.	Usual care from their primary care physicians	0.17	0.5	106	NR	66.2*	40.90%	NR	NR	NR	ACR/RR: 6mo
Hsieh et al. 2021[48]	Patients in cardiovascular outpatient department; Taiwan	Atrial Fibrillation; Cardiology	Participants accessed a web-based program with five domains: patient info, atrial fibrillation knowledge, anticoagulation instructions, symptom self-monitoring, and professional consultation, aiding disease tracking and treatment regulation.	Standard instructions including atrial fibrillation management manual and telephonic coaching on disease management.	0.5	2	232	NR	63.7*	52.0%	NR	NR	NR	ACR: 24mo
Indraratna et al. 2022[49]	Patients discharged after admission for either HF or ACS; Australia	HF or ACS; Cardiology	Usual Care plus TCC app and peripheral devices at discharge to measure vital signs. Data automatically transmitted, when readings fell outside predefined range monitoring team was alerted and course of action was decided.	Recommendation to follow up with the GP within 1 week of discharge and with the treating cardiologist and appropriate referrals.	NR	0.5	164	KIOLA	69*	27.7%	C:29(35); I:26(28)	C:29(35); I:32(34)	NR	ACR: 30d, 6mo
Jimenez et al. 2017[50]	Patients with HF with mid-range or preserved ejection fraction; Spain	HF; Cardiology	Telemedicine included daily signs and symptoms telemonitoring and structured follow-up by the means of video or audio-conference.	Usual Care including structured follow-up in the basis of face-to-face encounters	NR	0.5	116	NR	NR	42%	NR	NR	NR	CSR: 6mo (HF/cardiovascular)

© 2025 Pattar BSB et al. *JAMA Network Open*.

Johnson et al. 2022[51]	Patients with systolic or diastolic left ventricular HF; USA	HF; Cardiology	A secure website featuring educational videos, daily prompts, alerts for urgent issues, nurse monitoring, interactive symptom tracking, biometric feedback, and medication reminders.	Usual care consists of routine discharge planning that includes a review of the discharge medications and clinical discharge summary with the recommended follow-up.	NR	0.25	31	NR	60.1*	35.5%	C:34(68); I:30(60)	C:11(22); I:13(26)	NR	UR: 30d, 90d
Kant et al. 2024[52]	Patients planned for acute admission wards after ED presentation; Netherlands	Non-specific; Non-specific	Accelerometer-based sensors for continuous monitoring of heart rate, respiratory rate, activity, and posture. Data were available to physicians and nurses via a dashboard with trend analysis and Early Warning Score-like metrics, aiding discharge decisions.	Physicians decided on discharge during daily rounds using acute admission ward information, including medical history, medication use, and Early Warning Scores based on intermittently monitored vital signs.	DOH	0.08	400	Physician Dashboard: IntelliVue, Philips Electronics BV, Eindhoven, The Netherlands) ; Telemonitroing system: Healthdot, Philips Electronics BV, Eindhoven, The Netherlands	68.1*	41.1%	C:50(74); I:55(67)	C:33(49); I:39(48)	NR	UR: 30d
Khonsari et al. 2015[53]	Patients admitted with ACS: England	ACS; Cardiology	Participants received text-message reminders for medication intake and refills. SMS-based reminder system gathers, manages, and stores patient information to send text messages and record delivery reports.	Usual care for ACS post-discharge including cardiac rehabilitation and follow-up appointments with the cardiologist.	0.17	0.17	62	NR	C:60; I:58.5†	44.0%	NR	NR	NR	RR: 2mo
Kotooka et al. 2018[54]	Patients with HF; Japan	HF; Cardiology	Home telemonitoring system included a scale and sphygmomanometer. Nurses monitored data on a secure website, notifying physicians if thresholds were exceeded. Physicians could intervene as needed, with no restrictions.	Clinicians provided discharge education and encouraged the patients to measure their body weight by themselves every day.	0-2.58	0-2.58	181	Karada KarteTM Tanita Health-link Co. Ltd, Tokyo, Japan	C:80; I:88†	70.0%	NR	NR	C:1(20); I:0(0)	ACR
Kowalkowski et al. 2022[55]	Adult patients with high risk of readmission of mortality; Netherlands	Sepsis; Intensive Care	Multicomponent transition program using phone and EHR communication, including post-discharge medication review, symptom evaluation, comorbidity monitoring, and palliative care consultation.	Usual care included patient education, follow-up instructions, recommendations for primary care visits, and arrangements for care management.	NR	1	691	NR	80.7*	58.0%	NR	NR	NR	ACR/CO: 12mo§
Kraai et al. 2016[56]	Patients with documented reduced left ventricular ejection fraction; Netherlands	HF; Cardiology	Patients used a scale, blood pressure equipment, ECG device, and health monitor to record daily data. Deviations triggered a nurse call within two hours for symptom discussion.	Provided advice to the healthcare providers according to HF guidelines.	NR	0.75	177	NR	80.5*	71.5%	NR	NR	NR	ACR: 9mo
Krzowski et al. 2023[57]	Patients hospitalized due to MI; Poland	Acute MI; Cardiology	Rehabilitation supported by a mobile app providing educational resources, lifestyle recommendations, therapy adherence prompts, and vital sign reporting. Alerts were sent if data required clinic or emergency care.	Regular cardiac rehabilitation.	0.08	0.5	100	NR	73.6*	25.1%	NR	C:44(29); I:40(24)	C:10(7); I:14(8)	Comp¶
Kulshrestha et al. 2010[58]	Readmission history for cardiac-related reasons or ejection fraction; USA	HF; Cardiology	Patients measured vital signs and weight, transmitting data to a nurse. The nurse made weekly calls for instruction, monitored adherence, and addressed out-of-range readings or symptom changes.	Usual Care.	0.5	0.5	150	VitelNet	74.9*	67.7%	C:24(53); I:25(46)	C:28(62); I:23(43)	NR	ACR: 6mo
Lee et al. 2019[59]	Liver Transplant recipients; USA	Liver Transplant; Surgery	Telemedicine-based health management profile used a tablet and devices for recording vitals. Daily questions, reminders, and educational videos were provided. Data was reviewed and alerts addressed by a nurse coordinator.	Standard of care included discharge education and instruction to take and monitor vitals and when to contact office.	0.25	0.25	102	Epic Systems	58.9*	51.8%	NR	C:363(39) ; I:359(38)	NR	ACR: 30d, 90d
Levine et al. 2022[60]	Patients likely to return to community dwelling	Non-specific; General	Daily care from a home health aide, certified nursing assistant, nurse, and physician. Core technologies included	Patients transported to a traditional SNF and were not monitored in any way.	0.08	0.08	10	Health Tag, Spire Health	C:66; I:66†	78.10%	NR	NR	NR	ACR: 30d

© 2025 Pattar BSB et al. *JAMA Network Open*.

	status after SNF care; USA	medical wards	remote PT, automated medication dispensing, and continuous monitoring.											
Levine et al. 2022[61]	Patients that required admission and were acutely ill; USA	Non-specific; NR	Attending physician conducted an initial home visit, followed by daily video visits with nurse or paramedic assistance. Care included nurse visits, IV infusions, remote monitoring, respiratory therapies, and point-of-care testing.	The attending physician performed daily home rounds with a nurse/paramedic, plus twice-daily nurse visits.	0.08	0.08	172	VitalConnect	NR	NR	NR	NR	NR	RR: 30d§
Liang et al. 2021[62]	Inpatients with a high risk of readmission; USA	Non-specific; NR	Participants used wireless monitoring devices and a smartphone for communication. Nurses provided education, consultations, reminders, and conducted three home visits for care management.	Patients received discharge planning and home visits from nurses for care assessments, vital sign checks, education, and medication consultations.	0.5	0.5	200	NR	72.4*	38.60%	C:12(63); I:14(70)	C:7(37); I:9(45)	NR	RR: 6mo
Lisby et al. 2018[63]	Elderly patients in orthopedic department with at least 4 medications at admission; Denmark	Non-specific; Orthopedics	A clinical pharmacist and pharmacologist conducted a systematic medication review, including interviews with participants, medication reconciliation, and assessment for discrepancies, interactions, and inappropriate prescriptions.	A ward physician obtained medication history, performed review, and prescribed the in-hospital medication.	NR	0.25	108	NR	64*		NR	NR	NR	ACR: 90d
Lopez-Liria et al. 2019[64]	Patients who received implantation of a pacemaker; Switzerland	NR; Cardiology	Home monitoring utilized an internet-based service for Biotronik heart devices, transmitting data wirelessly. Physicians received automatic alerts for safety issues, managed by the implanting clinic.	Patients received standard hospital monitoring.	NR	1	50	Biotronik Home Monitoring® system	62.7*	40.7%	NR	NR	NR	ACR: 12mo
Lyng et al. 2012[65]	Patients hospitalized with HF; UK	HF; Cardiology	Patients used an electronic scale to transmit weight data wirelessly. An alarm triggered for weight gain over 2kg, prompting contact from the HF nurse to determine next steps.	Patients were recommended to weigh themselves daily and contact the HF clinic of weight gain of >2 kg in 3 days.	NR	1	344	Zenicor Medical Systems AB	63.6*	43.9%	NR	NR	NR	ACR/CO:12 mo
Madigan et al. 2013[66]	Patients hospitalized with HF; USA	HF; Cardiology	Telemonitoring at home prompted daily measurements of blood pressure, pulse, oxygen saturation, and weight. A nurse reviewed transmitted data, responding to abnormalities per agency protocol.	HF education included how to follow their own weights and symptoms. Home visits based on each agency's protocol and needs of the patient.	0.16	0.5	99	NR	64.5*	63.4%	NR	NR	NR	Time to CO
McWilliams et al. 2019[67]	Inpatients identified as high-risk for readmission; USA	Non-specific; NR	Nurses contacted patients before discharge and monitored progress via EMR. For 30 days post-discharge, patients accessed various healthcare providers and received weekly contact and coordinated care.	Usual care including follow-up appointment recommendations, discharge summaries to PCPs, home health service arrangements, and outreach like follow-up care management calls.	0.09	0.25	3710	NR	76.2*	54%	NR	NR	NR	ACR: 30d, 90d; RR: 60d
Mehta et al. 2020[68]	Patients aged 18 to 85 years scheduled to undergo hip or knee.; USA	Surgery; Surgery	Patients received a physical activity monitor, tracked daily pain scores via text messaging, received milestone updates, nonadherence alerts, and had access to clinicians through the Way to Health platform.	Usual care.	0.12	0.12	300	Way to Health platform	75.4*	57.4%	NR	NR	NR	RR: 45d
Minguez et al. 2014[69]	Patients hospitalized with COPD; Spain	COPD; NR	Patients had a discharge visit from the program at home, followed by telemonitoring installation. Daily vital signs and parameters were sent to the telemedicine platform for review by the pulmonologist.	Visit before discharge from the hospital and a discharge visit from the program at home daily nursing visits.	NR	0.5	30	NR	66.4*	59.0%	NR	NR	NR	RR: 30d, 90d, 6mo
Mizukawa et al. 2019[70]	Patients with an admission for HF; Japan	HF; Cardiology	Usual care plus 12-month disease management with telemonitoring. Patients received devices to monitor vitals, and nurses reviewed data. Abnormal readings prompted contact and further steps were determined.	Received a notebook to record daily self-monitoring vitals, education session using pre-existing booklet and HF treatment by physician.	1	2	39	Citizen systems co. ltd. & Cybercross Japan co. ltd.	C:50; I:41†	48.3%	NR	NR	NR	ACR: 24mo
Mousa et al. 2019[71]	Vascular surgery patients with groin incisions; USA	Vascular surgery; Surgery	Patients received a tablet and home monitoring devices that transmitted information to care managers who reviewed alerts, real-time patient data, and dialogue with the care team.	Routine discharge instructions and no monitoring.	NR	0.08	30	TeleMed 2020 (Indianapolis, Ind)	65.3*	62.5%	C:81(50); I:76(50)	NR	NR	RR: 30d
Nipp et al. 2019[72]	Patients admitted to oncology service with diagnosis of advanced cancer; England	Advanced cancer; Oncology	Patients reported symptoms daily via tablets, displayed during morning rounds. Alerts for symptom deviations guided	Participants reported symptoms each day using tablet computers. However, these patients' clinicians did	DOH	0.08	150	NR	C:73†	54.7%	C:278(56); I:538(55)	C:134(27); I:291(30)	C:124(25); I:250(26)	RR: 30d

© 2025 Pattar BSB et al. *JAMA Network Open*.

			oncology team decisions on symptom management based on clinical judgment.	not receive their symptom reports.										
Nipp et al. 2022[73]	Patients receiving treatment with palliative intent; USA	Advanced cancer; Oncology	Patients reported daily symptoms via tablets, providing numeric scores and alerts for worsening symptoms. Physicians received detailed reports during morning rounds, including symptom trajectories for the hospitalization.	Patients reported their symptoms each day however, clinical teams did not receive their symptom reports.	DOH	1	390	NR	73.9*	51.1%	C:151(66); I:95(73)	C:95(42); I:56(43)	NR	UR: 30d, 90d
Noel et al. 2020[74]	Patients discharged home with follow up care in Family or Internal Medicine clinical practices: USA	NR; NR	Patients provided with a smartphone, Bluetooth-enabled devices, and weekly virtual visits with a transition physician. Vital signs were measured daily, and medication adherence was assessed.	Review of patient instructions and discharge summary, encouraging follow-up with PCP within 7–14 days, and scheduling specialist appointments as needed.	0.08	1	102	NR	82.8*	60.6%	NR	NR	NR	ACR: 30d§
Ong et al. 2016[75]	HF patients with the initiation of or increase in diuretic treatment; USA	HF; Cardiology	Predischarge HF education, regularly scheduled telephone coaching, and home telemonitoring of weight, blood pressure, heart rate, and symptoms.	Usual care included robust predischarge education and often a post discharge follow-up telephone call.	0.08	0.5	1437	NR	58.9*	46.4%	NR	NR	NR	ACR/CO: 30d, 6mo
Pietrantonio et al. 2021[76]	Complex patients discharged from Internal Medicine; Switzerland	NR; Internal medicine	A portable wireless system enables continuous real-time vital signs monitoring, automatic NEWS score calculation, and personalized alerts via tablet or phone.	Standard of care with conventional monitoring.	0.01	0.08	110	WIN@Medical monitoring systems	56.3*	54.10%	NR	NR	NR	ACR: 30d
Pollak et al. 2019[77]	Patients with metastatic cancer, dementia, admission from a long-term care facility, or chronic illness; USA.	Non-specific; Not reported	EMR alerts prompted physicians to discuss goals of care for patients.	Standard of care.	NR	0.08	428	NR	78.1*	52.0%	NR	NR	NR	ACR: 30d
Polo et al. 2023[78]	African American or Hispanic persons hospitalized for a COPD; England	COPD; Not reported	Virtual pulmonary rehabilitation with continuous vital sign monitoring via Nonin watch. A pulmonologist was on-call during sessions for emergencies.	Standard pulmonary rehabilitation includes exercise equipment, vital sign monitors, supplemental oxygen, education lectures and support team.	0.15	0.5	266	NR	61*	28.3%	NR	NR	NR	CSR/CO: 6mo (COPD)
Pooni et al. 2023[79]	Patients undergoing elective colorectal surgery; Canada	Elective colorectal surgery; General Surgery	The Home to Stay app allows participants to report postoperative recovery, take photos of incisions, and access educational materials. Responses are monitored daily by physician assistants for tailored recommendations.	Patients received written instructions on complications and surgeon contact info, a follow-up call post-discharge, and an in-person visit with the surgeon.	0.08	0.08	282	Home to Stay app	76.5*	64.7%	NR	NR	NR	ACR: 30d
Prvu Bettger et al. 2020[80]	Total knee arthroplasty for the treatment of nontraumatic conditions; USA	Total Knee Arthroplasty; Orthopedics	The Virtual Exercise Rehabilitation Assistant is a cloud-based system using 3D tracking technology and an avatar coach for exercise guidance, providing feedback, and enabling telehealth visits with a therapist.	Patients followed care team's recommendations for all preoperative and postoperative medical and rehabilitative care.	NR	0.23	306	Virtual Exercise Rehabilitation Assistant (VERA; Reflexion Health)	64.2*	33.0%	NR	NR	NR	ACR: 3mo
Ravn-Nielsen et al. 2018[81]	Patients with a use of 5 or more prescribed drugs on a daily basis; Denmark	Non-specific; Acute admission wards	Conducted medication reconciliation via a pharmacist interview, communicated unresolved issues to the PCP, and followed up on medication changes three days later.	Usual care.	NR	0.5	1499	NR	C:58; I:57†	54.8%	C:59(41); I:67(46)	C:24(17); I:28(19)	NR	ACR: 30d, 6mo
Riegel et al. 2002[82]	Patients hospitalized with HF; USA	HF; Cardiology	Patients were contacted within 5 days post-discharge, with follow-up calls based on symptoms and needs. Shortness of breath prompted same-day calls to confirm physician instructions and medication access.	Usual Care.	0.5	0.5	358	Decision-support software program developed by Pfizer Inc.	69.8*	42.20%	NR	NR	NR	ACR: 90d, 6mo
Rosstad et al. 2017[83]	70 years or older and receive home care services after; Norway	Cardiac, infections, fractures, cancers, pulmonary diseases, and neurological diseases; Various	Used checklists in home care to monitor patients' health, social, and functional status, integrating them into EMR for improved follow-up and communication.	Usual Care.	0.08	1	308	NR	69.8*	61.5%	NR	NR	C:8(28); I:6(24)	UR: 30d§

© 2025 Pattar BSB et al. *JAMA Network Open*.

Rubin et al. 2022[84]	Established diagnosis of diabetes; USA	Non-specific (Previous diabetes diagnoses); NR	Standard care plus discharge instructions generated using EMR, patient-centered discharge education, HbA1c-based diabetes therapy adjustments, and post-discharge support.	Standard discharge instructions, education, medication reconciliation, and follow-up according to routine practice.	NR	0.08	93	Epic Hyperspace	58.7*	51.60%	NR	C:46(100); I:45(100)	NR	UR: 30d
Saleh et al. 2023[85]	Patients with emergency hospitalization for COPD exacerbation; Norway	COPD; Pulmonology	Telemedicine included a tablet for video calls with a respiratory nurse, oxygen saturation, and heart rate monitoring, with data securely transferred.	Best standard practice COPD care.	1	1	173	NR	34*	55.6%	NR	NR	NR	ACR: 12mo
Santana et al. 2017[86]	Patients with Multiple comorbidities and complicated medication profiles; Canada	Non-specific; Medical teaching unit	Electronic discharge communication tool generated and uploaded discharge summaries with structured content admission, hospital stay, treatments and follow-up care required.	Usual care included traditional discharge communication generated by dictation.	NR	0.25	1399	Epic Systems	54.6*	100.0%	NR	NR	NR	ACR/CO: 30d, 90d§
Schmaderer et al. 2022[87]	HF diagnosis or an episode of acute decompensated HF; USA	HF; Cardiology	The app provided reminders for weighing, medications, and appointments, with education, confidence building, and self-monitoring features. Virtual visits with health care provider on improving patient activation and self-management skills.	Training on the app for recording medications and weights. All app reminders and educational tips were disabled.	0.25	0.25	80	Play-It Health App	69.2*	56.4%	NR	NR	C:14(16); I:4(5)	ACR: 90d
Schwarz et al. 2008[88]	HF patients who were functionally impaired in at least 1 activity of daily living or one instrumental activity of daily living; USA	HF; Cardiology	The telemonitoring system measured weight daily and prompted participants to answer health-related questions. Clinical variances triggered nurse follow-ups, caregiver education, and physician notifications.	Usual care.	0.25	0.25	102	NR		43.3%	NR	NR	NR	CS UR: 90d (HF)
Somsiri et al. 2021[89]	Patients with HF; Thailand	HF; Cardiology	Discharge planning phase included HF-specific education, counseling, telemonitoring via an app to record vitals and symptoms and alerts, private messaging, and notifications of noncompliance or health risks.	Usual care included discharge planning, HF-specific education on drug use and self-care, and a nurse contact number for consultations.	0.12	0.15	146	NR	63.7*	52.0%	NR	C:164(48); I:172(47)	NR	ACR: 6w, 8w
Soran et al. 2008[90]	Evidence of systolic dysfunction via a left ventricular ejection fraction of ≤40%; USA	HF; Cardiology	A home-based program for daily HF monitoring, enabling timely interventions by physicians based on patient-reported symptoms and weight data.	Patients received a digital scale to weigh themselves daily and report HF symptoms, along with educational materials on when to seek medical attention.	NR	0.5	315	Alere Day Link Heart Failure Monitoring System	80.3*	52.4%	C:7(3); I:7(3)	C:41(15); I:36(14)	NR	ACR/CO: 6mo
Sorensen et al. 2020[91]	Patients with self-administered medication; Denmark	NR; Cardiology	Nurses assessed patients' medications for quantity and quality, comparing them with electronic prescriptions. Discrepancies were addressed with a doctor, and medications were organized for use or storage, with self-administration documented.	Hospital-provided medication during hospitalization was dispensed and administered by a nurse at each prescribed time point.	NR	0.08	250	NR	79.7*	72.5%	NR	NR	NR	ACR: 30d
Spaulding et al. 2022[92]	Adult candidates for inpatient elective operating procedures; USA	Inpatient elective operating procedures; Surgery	Patients used a digital tablet and monitoring devices to report vital signs and symptoms daily for 30 days. Alerts were triggered for abnormal readings or missed submissions, prompting nurse follow-ups.	Usual care included no monitoring.	0.08	0.08	292	NR	73*	46%	NR	NR	NR	ACR: 30d
Spierling Bagsic et al. 2023[93]	Hispanic adults with T2D and HbA1c ≥ 7.0 % in the last 30 days; USA	Non-specific; Non-specific	The Dulce Digital-COVID Aware program included pre-discharge diabetes education, blood glucose monitoring training with a cellular-connected device, and a digital texting platform for support and feedback on glucose levels.	Standard care for diabetes patients discharged, including pre-discharge education, training on blood glucose monitoring, and post-discharge support from a peer health coach.	0.49	0.49	172	Dulce Digital-COVID Aware	76.1*	27.6%	C:51(65); I:94(61)	NR	C:3(3); I:10(4)	ACR: 30d, 90d
Tamblyn et al. 2019[94]	Patients covered by provincial drug insurance; Canada	Non-specific; Medicine and Surgery	Medication reconciliation via EMR involved retrieving the community drug list at admission, reviewing hospital drugs during hospitalization, and faxing medication information to relevant physicians and pharmacies.	Usual care included medication reconciliation at admission and discharge.	DOH	0.25	3567	RAMQ data warehouse	87.5*	74.60%	NR	NR	NR	ACR: 30d, 90d§
Taylor et al. 2022[95]	Adult patients deemed high risk for readmission or mortality; USA	Sepsis; NR	Facilitates optimal care for sepsis survivors by identifying and treating new deficits, reviewing medications,	Usual transitional and outpatient care.	0.08	0.08	691	Atrium Health Community	65.5*	53.5%	NR	NR	NR	ACR/CO: 30d§

© 2025 Pattar BSB et al. *JAMA Network Open*.

			monitoring chronic conditions, and aligning care, including palliative options when necessary.					Connect (Epic Systems) and Enterprise Data Warehouse						
Tchalla et al. 2023[96]	Elderly patients with chronic diseases and discharged home; France	Chronic disease; Acute hospital care	Patients received education on a nurse-led telemonitoring program for 24/7 data transmission, with manual uploads for remote assessment and feedback from a geriatrician.	Usual care from their general practitioner including treating consequences of a fall but does not address patient risk behaviours.	1	1	534	e-GEROPASS software (algorithm-based software)	61*	35.5%	NR	NR	NR	UR: 12mo
Triller et al. 2007[97]	21 years or older with diagnosis of HF; USA	HF; NR	Usual care plus standardized clinical pharmacist services, with an initial comprehensive in-home medication assessment and two follow-up visits, during which physician notes and lab tests were reviewed.	Usual care.	0.06	0.5	154	Northeast health Health system	69.3*	1.3%	NR	NR	NR	ACR/CO: 6mo
van der Storm et al. 2024[98]	Adult patients who received ileostomy or colostomy; Netherlands	Ileostomy or colostomy; Surgery	Comprehensive app version featuring an information library, personalized timeline, notifications, instructional videos, and options to track weight, fluid intake, and stoma care while interacting anonymously with other patients.	Restricted version of the app that contained stoma-related information, lacking personalization and timing.	0.25	0.25	263	The Stoma App (NL-CA002-2020-53630)	72*	27.5%	NR	C:8(20); I:7(18)	C:10(26); I:9(22)	RR: 30d, 90d
Vianello et al. 2016[99]	Adult patients diagnosed with Class III-IV COPD; Italy	Adverse event; Outpatient pulmonary clinics	telemonitoring system with a pulse oximeter, transmitting heart rate and oxygen saturation data every other day or during clinical worsening for daily review and follow-up.	Usual care.	1	1	334	Wrist Clinic (TM) - Medical Concierge, Medic 4 all Italia, Italy	63.1*	18.5%	C:21(70); I:28(82)	C:4(13); I:11(32)	NR	CSR/ACR IR (COPD)
Villani et al. 2014[100]	Patients with HF hospitalized with high risk of readmission; Italy	HF; Cardiology	Telemonitoring web-based system assisting with clinical decisions. Cardiologists set monitoring variables and frequency, analyze data, and communicate therapy adjustments or consultations as needed.	Patients were discharged with appointments for follow-up every 3 months in accordance with guidelines for fragile patients.	1	1	80	NR	76*	35.5%	NR	NR	NR	ACR: 12mo
Visade et al. 2022[101]	Patients at least 75 years old hospitalized in an acute geriatric unit; France	Non-specific; Acute geriatric unit	A multi-interventional approach included entry medication review by a pharmacist; structured assessment with a psychiatrist; discharge review; and secure data transmission to healthcare professionals.	Usual care involved no in/out case management or medication review; the hospital pharmacist conducted a level 2 pharmaceutical analysis.	NR	0.08	108	NR	70.6*	43.7%	NR	C:288(31); I:140(19)	NR	ACR/RR: 30d
Visperas et al. 2021[102]	Patients undergoing total hip or knee arthroplasty; USA	Elective total hip or knee arthroplasty; Orthopedics	Patients receive key information on surgery preparation, medication, postoperative recovery, and rehabilitation, with follow-ups on pain levels and recovery metrics to ensure timely care.	Usual care included a preoperative education class about procedure.	0.25	0.25	514	Stryker Performance Solutions JointCOACH (web-based IPSP)	60.6*	27.6%	NR	NR	NR	ACR: 30d, 90d
Volpp et al. 2017[103]	Hospitalized 18-80 year old patients, prescribed at least 2 study medications and discharged home; USA	MI; NR	Participants received up to four electronic pill bottles, daily lottery incentives for adherence, support from friends or family, access to social work resources, and close monitoring from a staff engagement advisor.	Usual care.	1	1	1509	Vitality GlowCaps bottle transmitted to Way to Health	66.5*	25.0%	NR	NR	NR	ACR: 12mo§
Wakefield et al. 2008[104]	Patients admitted for a possible HF exacerbation and communication deficits; USA	HF; NR	Patients were contacted 14 times over three months post-discharge. They received tools for self-monitoring HF symptoms, and nurses reinforced care plans and coordinated adjustments based on patient data.	Usual discharge teaching, follow-up clinic appointments, and contact with primary care nurse case manager by telephone.	0.25	1	148	NR	C:60; I:63†	45.3%	NR	NR	NR	ACR/RR: 12mo§
Weiss et al. 2019[105]	Inpatient adults discharged home; USA	Non-specific; Medical surgical units	Using the 8-item Readiness for Hospital Discharge Scale, 33 intervention units implemented protocols.	Usual care.	DOH	0.08	144 868	NR	51*	31.6%	C:52(72); I:57(71)	C:43(60); I:47(59)	C:35(49); I:37(46)	ACR: 30d
Wen et al. 2024[106]	Met diagnostic criteria for CHD; China	Coronary heart disease; Cardiology	A linked health platform allows doctors to adjust care, nurses to manage chronic diseases, and securely encrypts patient	Traditional discharge, with nurses providing health education and guiding settlement. Nurses followed	1.5	1.5	560	NR	60.6*	31.8%	C:50(83); I:42(78)	C:27(45); I:36(67)	NR	Time to readmission

© 2025 Pattar BSB et al. *JAMA Network Open*.

			data, enabling online communication and data export via the hospital interface.	up by phone three days post-discharge.										
Widmer et al. 2017[107]	Patients undergoing cardiac rehabilitation following ACS intervention; USA	Acute coronary intervention; Cardiology	Digital health intervention includes reporting of dietary and exercise habits and educational information.	Usual care.	0.25	0.25	80	Healarium, inc	57.3*	14.5%	C:301(60); I:321(64)	C:196(39); I:200(40)	C:17(3); I:14(3)	ACR: 90d, 6mo
Wilson et al. 2023[108]	Patients hospitalized to unit labeled high risk of needing Palliative care services in the next 7 days; USA	NR; Cardiology, critical care or oncology	The control tower/acute care multi-patient system analyzes EMR data to predict palliative care needs, selecting the top 12 patients for daily review by palliative care clinicians.	If a patient was identified to have a need, the palliative care specialist discussed with bedside provider and the patient was seen.	DOH	0.25	2544	Control Tower	72.2*	51.0%	NR	NR	NR	ACR: 30d, 60d, 90d
Wolf et al. 2016[109]	Younger than 75, admitted for suspected ACS and subsequently diagnosed of MI or unstable angina pectoris; Sweden	ACS; Cardiology	a mobile app with fatigue tracking, a symptom trend graph, and activity monitoring, along with a webpage offering symptom tracking, diary entries, chat, and resource links.	Usual care.	0.17	0.5	199	NR	C:82; I:71.5†	30.7%	NR	NR	NR	ACR: 6mo
Woodend et al. 2008[110]	Patients with symptomatic HF or angina; Canada	HF or angina; Cardiology	Weekly video conferences with a nurse, daily transmissions of weight and blood pressure, and periodic 12-lead ECGs to monitor progress and provide self-care education.	Usual care provided to patients with angina or HF discharged from the hospital.	0.25	1	249	NR	NR	48.2%	C:29(63); I:27(63)	C:6(13); I:6(14)	NR	No. of ACR per patient: 90d, 1y
Yap et al. 2022[111]	Adult patients discharge with 3 or more regular medications and more scheduled pharmacy medication fill visit; Malaysia	NR; NR	Pharmacist-assisted intervention included medication reconciliation at discharge, bedside delivery of medications with counseling, and post-discharge follow-up calls for medication instructions and refill reminders.	Routine hospital discharge process and discharge medications were managed by the respective medical wards.	NR	0.08	168	NR	71*	33.5%	C:57(63); I:55(60)	C:34(37); I:31(34)	NR	ACR: 30d
Yin et al. 2019[112]	Patients with chronic HF; China	HF; Cardiology	Patients received a Weight Intervention Handbook pre-discharge, which included guidance on weight monitoring, exercise, and diet, along with follow-up via a WeChat group and quarterly health education.	Usual medication guidance before discharge.	1	1	103	WeChat	56.4*	48.2%	NR	NR	NR	No. of ACR: 12mo
You et al. 2020[113]	Patients hospitalized with exacerbation of HF and discharged home; China	HF; Cardiology	Patients received standard care plus a 14-day post-discharge program, including nurse-specialist phone calls to assess symptoms, weight changes, and medication adherence, providing immediate feedback on next steps.	Standard care including disease status explanation, adherence to medications, low-salt diet guidance, fluid restriction, and daily body weight monitoring by ward nurses at discharge.	0.04	0.23	158	NR	65.4*	59.7%	NR	NR	NR	ACR: 12w
Yu et al. 2020[114]	Adult patients who underwent a coronary artery bypass graft and prescribed at least one oral medication; China	Coronary artery bypass graft; NR	Participants used an app with medication reminders, cardiac education, and weekly questionnaires to track adherence and receive feedback on their secondary prevention goals.	Standard care including cardiology education, instruction on secondary prevention, and promotion of self-care management from nurses and physicians during inpatient stay.	0.5	0.5	1000	Heart health application	69.7*	31.6%	NR	NR	NR	ACR: 6mo
Zhao et al. 2004[115]	60 or older, admitted with a diagnosis of angina or MI and discharged home; China	Angina or MI; Cardiology	Discharge planning, home visits, and telephone follow-ups after discharge, recording daily heart discomfort for four weeks using the Borg Scale for Rating Perceived Exertion.	Routine care.	0.08	0.23	200	NR	57.8*	48.3%	NR	NR	NR	CSR: 4w (cardiac)
Zisis et al. 2021[116]	18 years or older admitted for acute decompensated HF; Australia	HF; Cardiology or general medicine wards	App engages patients daily with education, prompts for daily weight tracking, and assesses HF knowledge and self-care efficacy at the start and end.	Usual care (did not enroll to app).	0.15	0.08	36	Heart Failure app	C:65; I:67†	49.0%	NR	NR	NR	ACR: 30d, 90d

Notes. *: Overall mean age; †: Median age; ‡: Composite of all-cause rehospitalization, emergency department visits, or mortality; §: Adjusted Outcomes; ¶: Need for rehospitalization and/or urgent outpatient care. Abbreviations: ACR, all-cause readmission; ACS, acute coronary syndrome; C, comparison; CGA, Comprehensive Geriatric Assessment; CO, compositive outcomes; COPD, chronic obstructive pulmonary disease; CS, cause specific; CSR, cause specific readmission; DOH, duration of hospitalization; ECG, electrocardiogram; EMR, electronic medical record; GP, general practitioner; HF, heart failure; I, intervention; IBD, inflammatory bowel disease; IR, incidence rate; MI, myocardial

© 2025 Pattar BSB et al. *JAMA Network Open*.

infarction; MTM, medication therapy management; NP; nurse practitioner; NR, not reported; PCP, primary care physician; PT, physical therapy; RN, registered nurse; RR, readmission rate; SNF, skilled nursing facility; UC, usual care; UR, unplanned readmissions; VAD, ventricular assist device.

© 2025 Pattar BSB et al. *JAMA Network Open*.

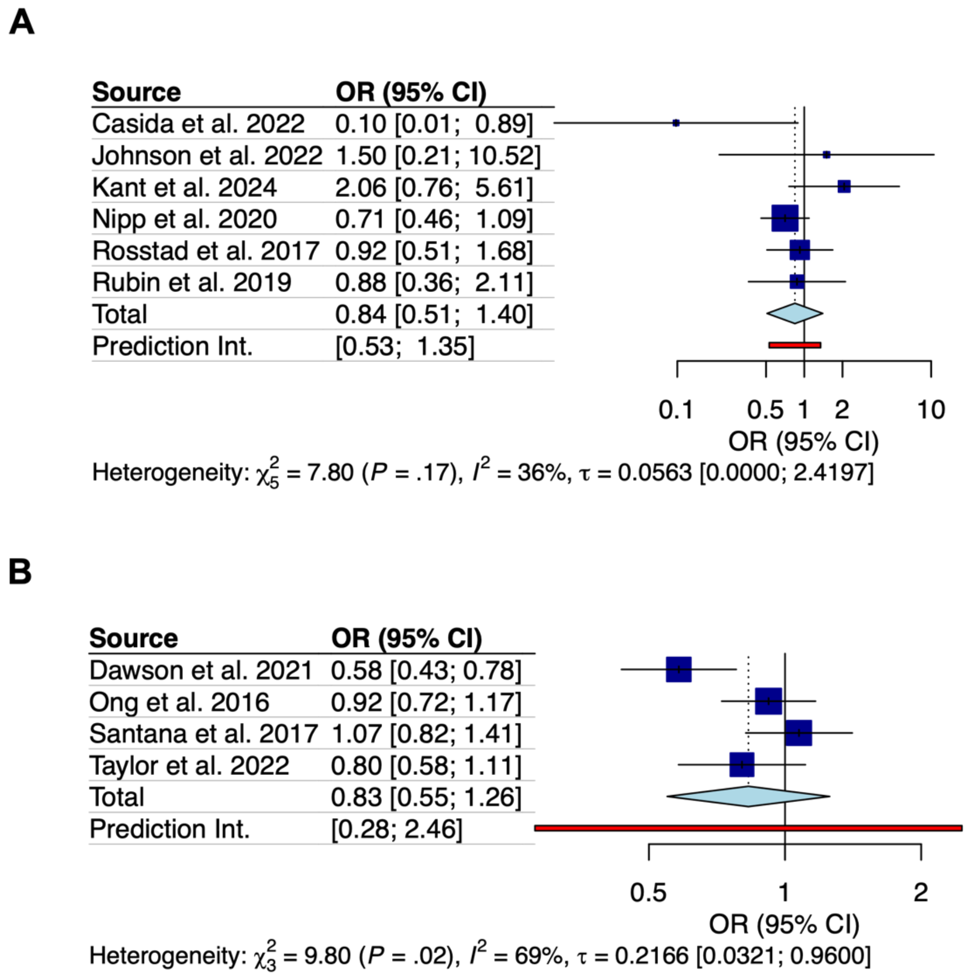

eFigure 1. Summary of meta-analysis for odds ratios of 30-day secondary outcomes. **A**: Unplanned Readmission; **B**: Composite Outcome. Effect estimates <1 favour EHR-based interventions in reducing readmissions. Effect estimates >1 favour the control, without an EHR-based intervention, in reducing readmissions. Abbreviations: CI: Confidence Interval. OR: Odds Ratio.

© 2025 Pattar BSB et al. *JAMA Network Open*.

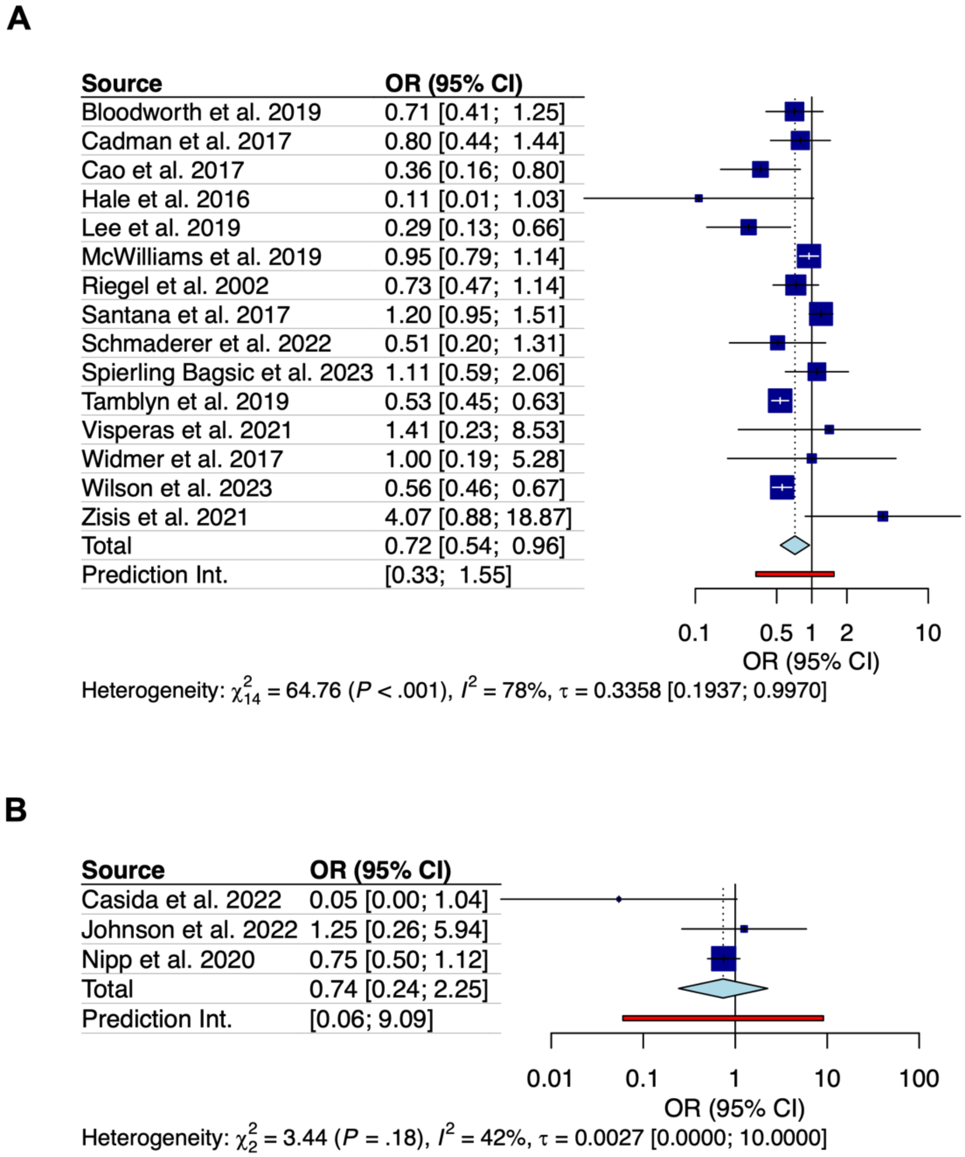

eFigure 2. Summary of meta-analysis for odds ratios of 90-day secondary outcomes. **A**: All-Cause Readmission; **B**: Unplanned Readmission. Meta-analysis for 90-day composite outcome not completed due to insufficient number of studies. Effect estimates <1 favour EHR-based interventions in reducing readmissions. Effect estimates >1 favour the control, without an EHR-based intervention, in reducing readmissions. Abbreviations: CI: Confidence Interval. OR: Odds Ratio.

© 2025 Pattar BSB et al. *JAMA Network Open*.

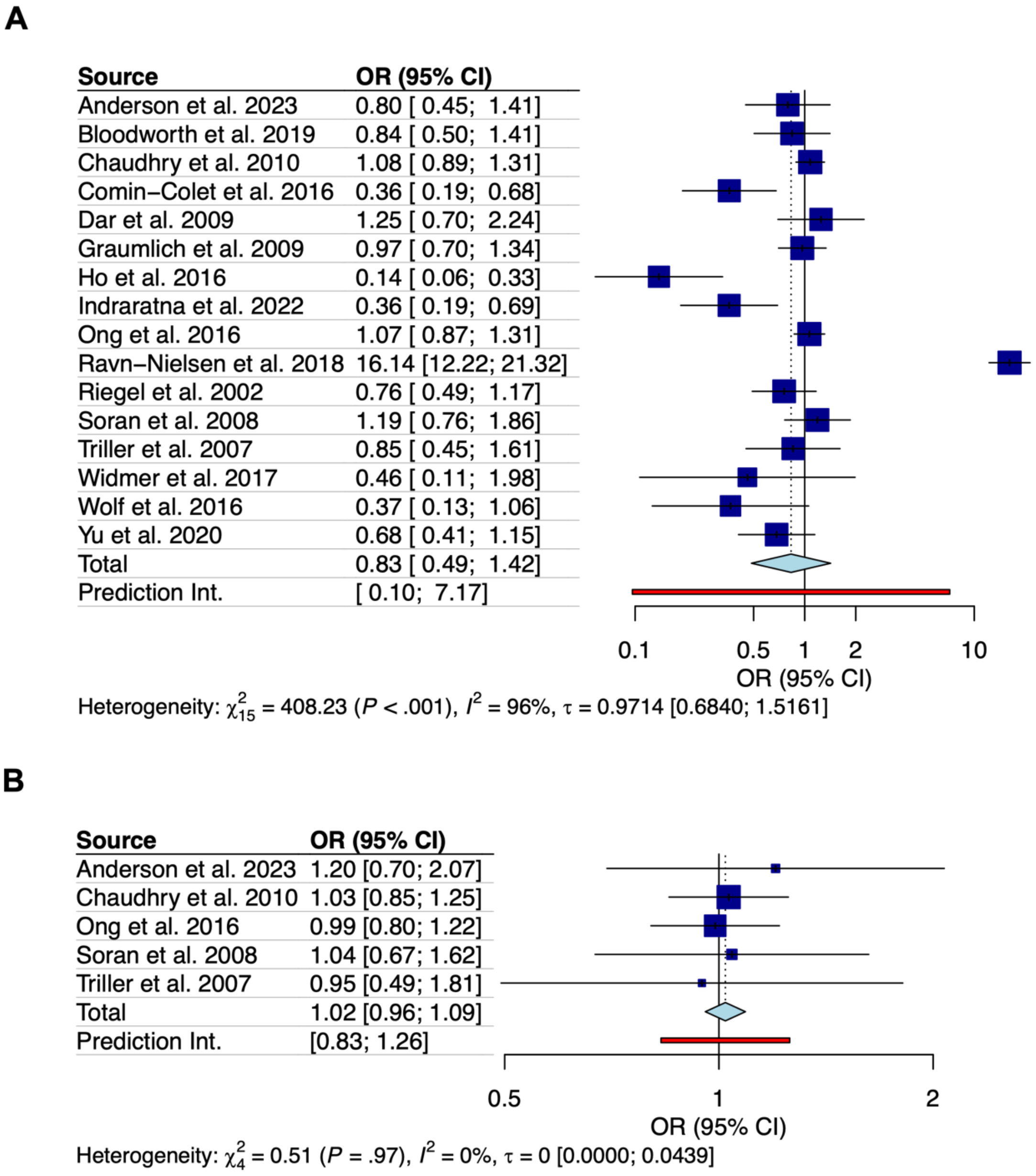

eFigure 3. Summary of meta-analysis for odds ratios of 6-month secondary outcomes. **A**: All-Cause Readmission; **B**: Composite Outcome. Meta-analysis for 6-month unplanned readmissions not completed due to insufficient number of studies. Effect estimates <1 favour EHR-based interventions in reducing readmissions. Effect estimates >1 favour the control, without an EHR-based intervention, in reducing readmissions. Abbreviations: CI: Confidence Interval. OR: Odds Ratio.

© 2025 Pattar BSB et al. *JAMA Network Open*.

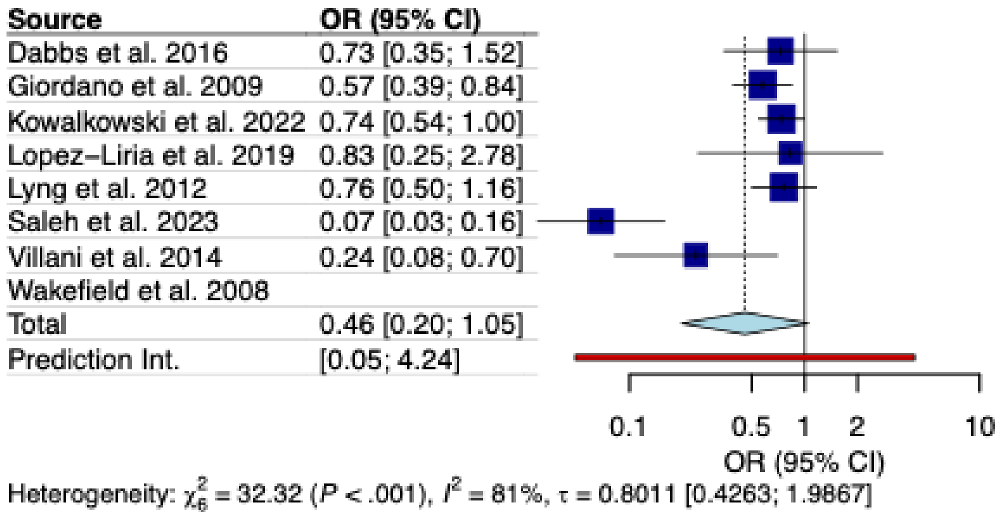

eFigure 4. Summary of meta-analysis for odds ratios of 12-month all cause readmissions. Meta-analysis for 12-month unplanned readmission and composite outcome not completed due to insufficient number of studies. Effect estimates <1 favour EHR-based interventions in reducing readmissions. Effect estimates >1 favour the control, without an EHR-based intervention, in reducing readmissions. Abbreviations: CI: Confidence Interval. OR: Odds Ratio.

© 2025 Pattar BSB et al. *JAMA Network Open*.

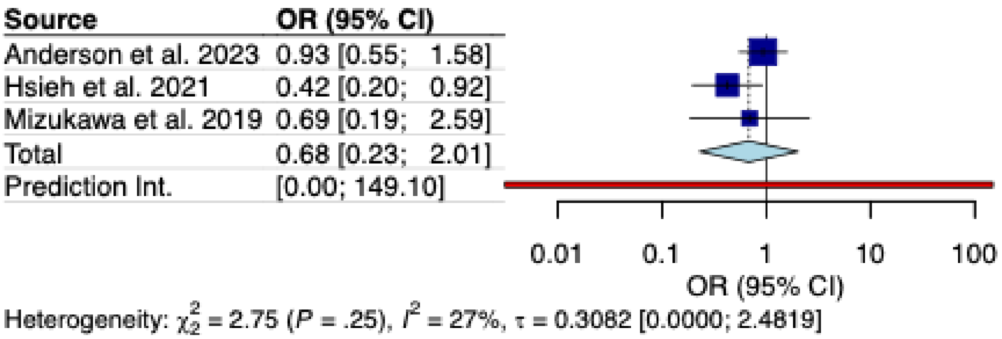

eFigure 5. Summary of meta-analysis for odds ratios of 24-month all cause readmissions. Meta-analysis for 24-month unplanned and composite outcome not completed due to insufficient number of studies. Effect estimates <1 favour EHR-based interventions in reducing readmissions. Effect estimates >1 favour the control, without an EHR-based intervention, in reducing readmissions. Abbreviations: CI: Confidence Interval. OR: Odds Ratio.

© 2025 Pattar BSB et al. *JAMA Network Open*.

eTable 5. Revised Cochrane Risk-of-Bias Tool for Randomized Controlled Trials, excluding conference abstracts (n=3).

	Risk of bias domains					
	D1	D2	D3	D4	D5	Overall
Adamson et al. 2016	+	+	+	-	+	-
Ahmad et al. 2022	-	-	+	X	+	X
Altfeld et al. 2013	+	+	+	+	+	+
Anderson et al. 2023	+	-	+	+	+	-
Asch et al. 2022	+	-	+	+	+	-
Balaban et al. 2008	-	-	+	X	+	X
Benatar et al. 2003	+	+	+	+	+	+
Bentley et al. 2014	+	+	+	+	+	+
Bloodworth et al. 2019	+	+	+	+	+	-
Blum et al. 2014	+	+	+	+	+	+
Bonnet-Zamponi et al. 2013	+	+	+	+	+	+
Bowles et al. 2011	+	+	+	+	+	+
Boxer et al. 2022	+	+	+	-	+	-
Breathett et al. 2018	+	+	+	+	+	+
Bressman et al. 2024	+	+	+	+	+	+
Broadbent et al. 2018	+	+	+	+	+	+
Cadman et al. 2017	+	X	+	-	+	X
Cao et al. 2017	+	+	+	+	+	+
Casida et al. 2022	+	+	+	+	+	+
Ceschi et al. 2021	+	-	+	+	+	-
Chau et al. 2012	+	+	+	-	+	-
Chaudhry et al. 2010	+	+	+	+	+	+
Cleland et al. 2005	+	+	+	+	+	+
Comin-Colet et al. 2016	+	+	+	+	+	+
Cossette et al. 2017	+	+	+	+	+	+
Creber et al. 2019	+	+	+	+	+	+
Dabbs et al. 2016	+	+	+	+	+	+
Dar et al. 2009	+	+	+	+	+	+
Dawson et al. 2021	+	+	+	+	+	+
Dendale et al. 2012	+	+	+	+	+	+
Downey et al. 2020	+	+	+	+	+	+
Finn et al. 2011	+	+	+	+	+	+
Franchi et al. 2016	+	+	+	+	+	+
Frederix et al. 2015	+	+	+	+	+	+
Gallagher et al. 2017	+	+	+	+	+	+
Giordano et al. 2009	-	+	+	+	+	-
Goldberg et al. 2003	+	+	+	+	+	+
Goldman et al. 2014	+	+	+	+	+	+
Graumlich et al. 2009	+	+	+	+	+	+
Gurwitz et al. 2014	+	X	+	+	+	X
Haag et al. 2016	+	-	+	+	+	-
Habib et al. 2021	-	+	+	+	+	-
Hajizadeh et al. 2020	+	X	+	+	+	X
Hale et al. 2016	-	+	+	+	+	-
Heaton et al. 2019	-	X	+	+	-	X
Ho et al. 2016	+	+	+	+	+	+
Hsieh et al. 2021	+	+	+	+	+	+
Indraratna et al. 2022	+	+	+	+	+	+
Johnson et al. 2022	+	+	+	+	+	+
Kant et al. 2024	+	+	+	+	+	+
Khonsari et al. 2015	+	+	+	+	+	+
Kotooka et al. 2018	+	+	+	-	+	-
Kowalkowski et al. 2022	+	-	+	+	+	-
Kraii et al. 2016	+	-	+	+	+	-
Krzowski et al. 2023	+	+	+	+	X	X
Kulshrestha et al. 2010	-	+	+	+	X	X
Lee et al. 2019	+	+	+	+	+	+
Levine et al. 2022	+	+	+	+	+	+
Levine et al. 2022*	+	+	+	+	+	+
Liang et al. 2021	+	+	+	+	+	+
Lisby et al. 2018	+	+	+	+	+	+
Lopez-Liria et al. 2019	+	+	+	+	+	+
Lyng et al. 2012	-	+	+	+	+	-
Madigan et al. 2013	+	+	+	+	+	+
McWilliams et al. 2019	+	+	+	+	+	+
Mehta et al. 2020	+	+	+	+	+	+
Mizukawa et al. 2019	+	+	+	+	+	+
Nipp et al. 2019	+	+	+	+	+	+
Nipp et al. 2022	+	+	+	+	+	+
Noel et al. 2020	+	+	+	+	+	+
Ong et al. 2016	+	+	+	+	+	+
Pietrantonio et al. 2021	+	+	+	+	+	+
Pollak et al. 2019	+	+	+	-	+	-
Polo et al. 2023	+	+	+	+	+	+
Pooni et al. 2023	-	+	+	+	+	-
Prvu Bettger et al. 2020	+	+	+	+	+	+
Ravn-Nielsen et al. 2018	+	+	+	+	+	+
Riegel et al. 2002	-	+	+	+	+	-
Rosstad et al. 2017	-	+	+	+	+	-
Rubin et al. 2022	+	+	+	+	+	+
Saleh et al. 2023	+	+	+	+	+	+
Santana et al. 2017	+	+	+	+	+	+
Schmaderer et al. 2022	+	+	+	+	+	+
Schwarz et al. 2008	+	+	+	+	+	+
Somsiri et al. 2021	+	+	+	+	+	+
Soran et al. 2008	+	+	+	+	+	+
Sorensen et al. 2020	-	+	+	+	+	-
Spaulding et al. 2022	-	+	+	-	+	-

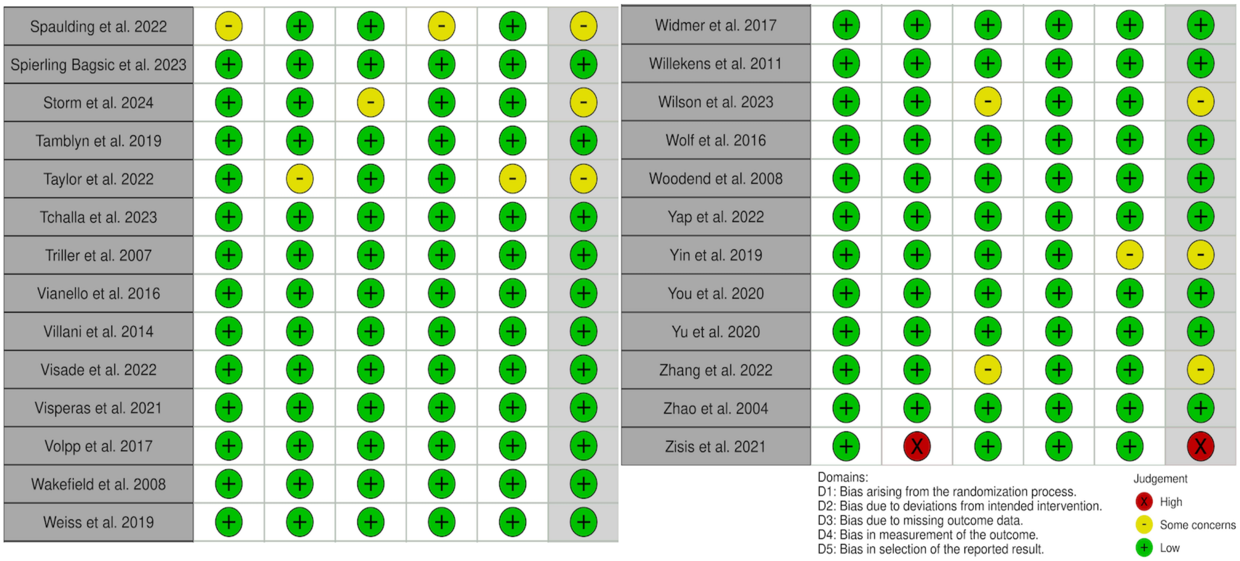

Notes. *: Refers to citation 61.

© 2025 Pattar BSB et al. *JAMA Network Open*.

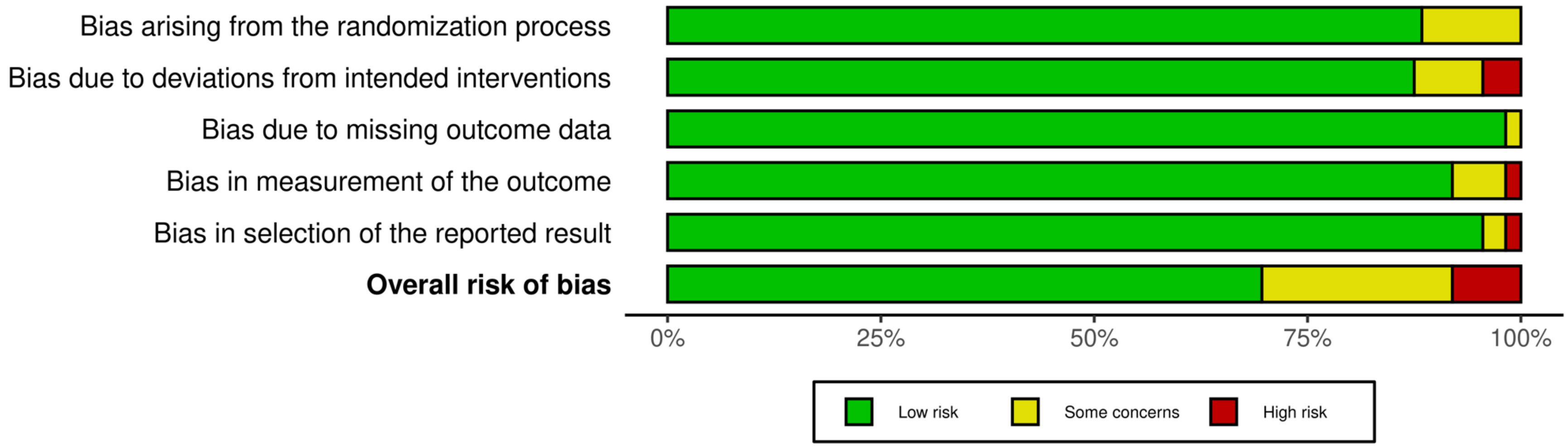

eFigure 6. Assessment of quality of included studies, excluding conference abstracts (n=3), using the revised Cochrane 'Risk of Bias' tool for Randomized Clinical Trials.

© 2025 Pattar BSB et al. *JAMA Network Open*.

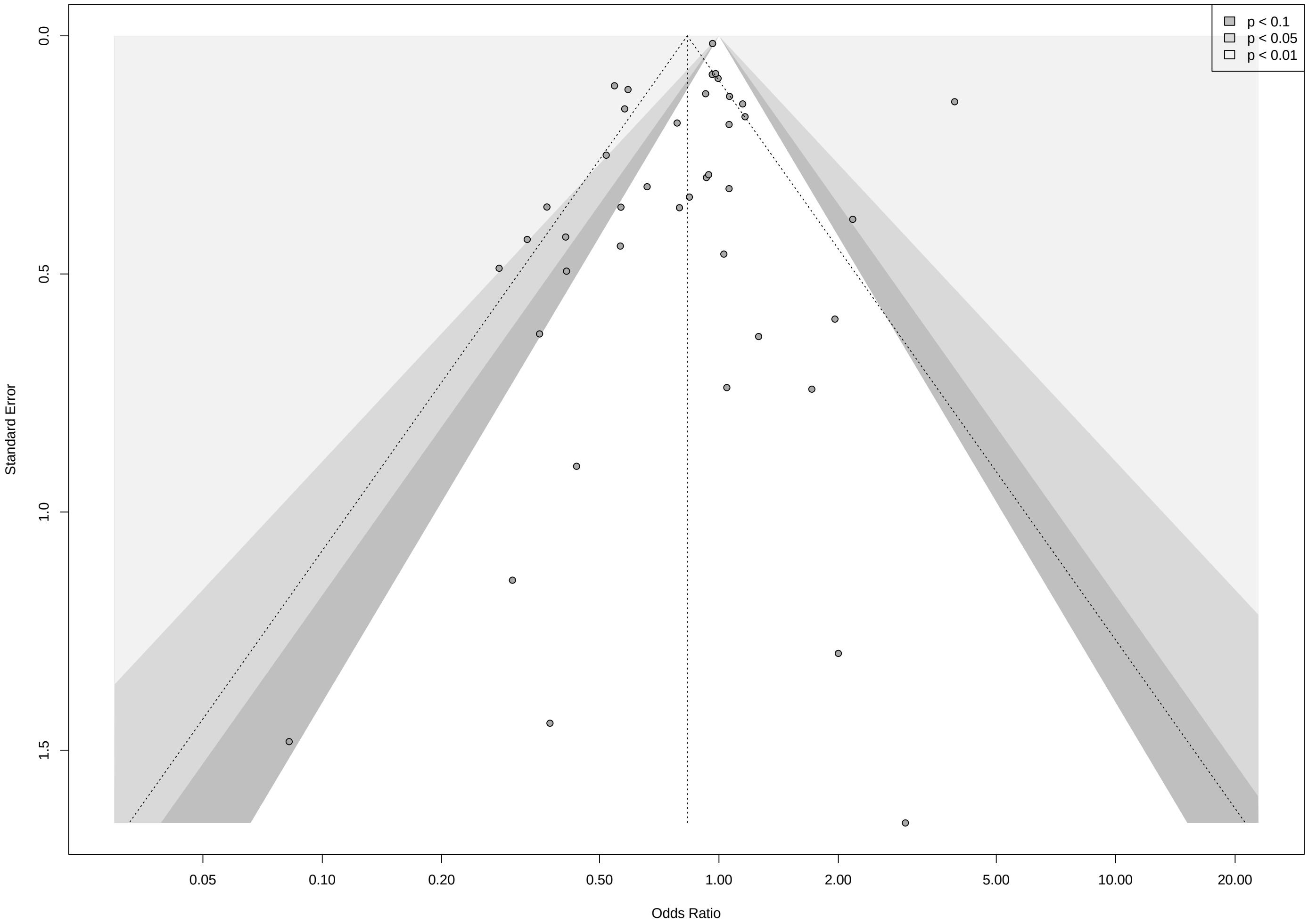

eFigure 7. Funnel plot of publication bias.

© 2025 Pattar BSB et al. *JAMA Network Open*.

Appendix. Reference list of included studies.

1. Adamson PB, Abraham WT, Stevenson LW, et al. Pulmonary Artery Pressure-Guided Heart Failure Management Reduces 30-Day Readmissions. *Circ Heart Fail.* 2016;9(6).
2. Ahmad T, Desai NR, Yamamoto Y, et al. Alerting Clinicians to 1-Year Mortality Risk in Patients Hospitalized With Heart Failure: The REVEAL-HF Randomized Clinical Trial. *JAMA Cardiol.* 2022;7(9):905-912.
3. Andersen FD, Trolle C, Pedersen AR, et al. Effect of telemonitoring on readmissions for acute exacerbation of chronic obstructive pulmonary disease: A randomized clinical trial. *J Telemed Telecare.* 2023;30(9):1417-1424.
4. Asch DA, Troxel AB, Goldberg LR, et al. Remote Monitoring and Behavioral Economics in Managing Heart Failure in Patients Discharged From the Hospital: A Randomized Clinical Trial. *JAMA Intern Med.* 2022;182(6):643-649.
5. Altfeld SJ, Shier GE, Rooney M, et al. Effects of an enhanced discharge planning intervention for hospitalized older adults: a randomized trial. *Gerontologist.* 2013;53(3):430-440.
6. Balaban RB, Weissman JS, Samuel PA, Woolhandler S. Redefining and redesigning hospital discharge to enhance patient care: a randomized controlled study. *J Gen Intern Med.* 2008;23(8):1228-1233.
7. Benatar D, Bondmass M, Ghitelman J, Avitall B. Outcomes of chronic heart failure. *Arch Intern Med.* 2003;163(3):347-352.
8. Bentley CL, Mountain GA, Thompson J, et al. A pilot randomised controlled trial of a Telehealth intervention in patients with chronic obstructive pulmonary disease: challenges of clinician-led data collection. *Trials.* 2014;15:313.
9. Bloodworth LS, Malinowski SS, Lirette ST, Ross LA. Pharmacist linkage in care transitions: From academic medical center to community. *J Am Pharm Assoc (2003).* 2019;59(6):896-904.
10. Blum K, Gottlieb SS. The effect of a randomized trial of home telemonitoring on medical costs, 30-day readmissions, mortality, and health-related quality of life in a cohort of community-dwelling heart failure patients. *J Card Fail.* 2014;20(7):513-521.
11. Bonnet-Zamponi D, d'Arailh L, Konrat C, et al. Drug-related readmissions to medical units of older adults discharged from acute geriatric units: results of the Optimization of Medication in AGEd multicenter randomized controlled trial. *J Am Geriatr Soc.* 2013;61(1):113-121.
12. Bowles KH, Hanlon AL, Glick HA, et al. Clinical effectiveness, access to, and satisfaction with care using a telehomecare substitution intervention: a randomized controlled trial. *Int J Telemed Appl.* 2011;2011:540138.
13. Boxer RS, Dolansky MA, Chaussee EL, et al. A Randomized Controlled Trial of Heart Failure Disease Management in Skilled Nursing Facilities. *J Am Med Dir Assoc.* 2022;23(3):359-366.
14. Breathett K, Maffett S, Foraker RE, et al. Pilot Randomized Controlled Trial to Reduce Readmission for Heart Failure Using Novel Tablet and Nurse Practitioner Education. *Am J Med.* 2018;131(8):974-978.

© 2025 Pattar BSB et al. *JAMA Network Open*.

15. Bressman E, Long JA, Burke RE, et al. Automated Text Message–Based Program and Use of Acute Health Care Resources After Hospital Discharge: A Randomized Clinical Trial. *JAMA Network Open.* 2024;7(4):e243701-e243701.
16. Broadbent E, Garrett J, Jepsen N, et al. Using Robots at Home to Support Patients With Chronic Obstructive Pulmonary Disease: Pilot Randomized Controlled Trial. *J Med Internet Res.* 2018;20(2):e45.
17. Cadman B, Wright D, Bale A, et al. Pharmacist provided medicines reconciliation within 24 hours of admission and on discharge: a randomised controlled pilot study. *BMJ Open.* 2017;7(3):e013647.
18. Cao XY, Tian L, Chen L, Jiang XL. Effects of a hospital-community partnership transitional program in patients with coronary heart disease in Chengdu, China: A randomized controlled trial. *Jpn J Nurs Sci.* 2017;14(4):320-331.
19. Casida JM, Pavol M, Budhathoki C, et al. A pilot clinical trial of a self-management intervention in patients with a left ventricular assist device. *J Artif Organs.* 2022;25(2):91-104.
20. Ceschi A, Noseda R, Pironi M, et al. Effect of Medication Reconciliation at Hospital Admission on 30-Day Returns to Hospital: A Randomized Clinical Trial. *JAMA Netw Open.* 2021;4(9):e2124672.
21. Chau JP, Lee DT, Yu DS, et al. A feasibility study to investigate the acceptability and potential effectiveness of a telecare service for older people with chronic obstructive pulmonary disease. *Int J Med Inform.* 2012;81(10):674-682.
22. Chaudhry SI, Mattera JA, Curtis JP, et al. Telemonitoring in patients with heart failure. *N Engl J Med.* 2010;363(24):2301-2309.
23. Cleland JG, Louis AA, Rigby AS, Janssens U, Balk AH. Noninvasive home telemonitoring for patients with heart failure at high risk of recurrent admission and death: the Trans-European Network-Home-Care Management System (TEN-HMS) study. *Journal of the American College of Cardiology.* 2005;45(10):1654-1664.
24. Comín-Colet J, Enjuanes C, Verdú-Rotellar JM, et al. Impact on clinical events and healthcare costs of adding telemedicine to multidisciplinary disease management programmes for heart failure: Results of a randomized controlled trial. *J Telemed Telecare.* 2016;22(5):282-295.
25. Cossette B, Éthier JF, Joly-Mischlich T, et al. Reduction in targeted potentially inappropriate medication use in elderly inpatients: a pragmatic randomized controlled trial. *Eur J Clin Pharmacol.* 2016;73(10):1237-1245.
26. Creber RM, Grossman LV, Ryan B, et al. Engaging hospitalized patients with personalized health information: a randomized trial of an inpatient portal. *J Am Med Inform Assoc.* 2019;26(2):115-123.
27. DeVito Dabbs A, Song MK, Myers BA, et al. A Randomized Controlled Trial of a Mobile Health Intervention to Promote Self-Management After Lung Transplantation. *Am J Transplant.* 2016;16(7):2172-2180.
28. Dang F, Habashi P, Gallinger Z, Nguyen G. Sa1616: PRICE: PREVENTING READMISSIONS IN IBD CENTRES OF EXCELLENCE. *Gastroenterology.* 2022;162(7, Supplement):S-441.

© 2025 Pattar BSB et al. *JAMA Network Open*.

29. Dar O, Riley J, Chapman C, et al. A randomized trial of home telemonitoring in a typical elderly heart failure population in North West London: results of the Home-HF study. *Eur J Heart Fail.* 2009;11(3):319-325.
30. Dawson NL, Hull BP, Vijapura P, et al. Home Telemonitoring to Reduce Readmission of High-Risk Patients: a Modified Intention-to-Treat Randomized Clinical Trial. *J Gen Intern Med.* 2021;36(11):3395-3401.
31. Dendale P, De Keulenaer G, Troisfontaines P, et al. Effect of a telemonitoring-facilitated collaboration between general practitioner and heart failure clinic on mortality and rehospitalization rates in severe heart failure: the TEMA-HF 1 (TElemonitoring in the MAnagement of Heart Failure) study. *Eur J Heart Fail.* 2012;14(3):333-340.
32. Downey CL, Croft J, Ainsworth G, et al. Trial of remote continuous versus intermittent NEWS monitoring after major surgery (TRaCINg): a feasibility randomised controlled trial. *Pilot Feasibility Stud.* 2020;6(1):183.
33. Finn KM, Heffner R, Chang Y, et al. Improving the discharge process by embedding a discharge facilitator in a resident team. *J Hosp Med.* 2011;6(9):494-500.
34. Franchi C, Tettamanti M, Djade CD, et al. E-learning in order to improve drug prescription for hospitalized older patients: a cluster-randomized controlled study. *Br J Clin Pharmacol.* 2016;82(1):53-63.
35. Frederix I, Van Driessche N, Hansen D, et al. Increasing the medium-term clinical benefits of hospital-based cardiac rehabilitation by physical activity telemonitoring in coronary artery disease patients. *Eur J Prev Cardiol.* 2015;22(2):150-158.
36. Gallagher BD, Moise N, Haerizadeh M, Ye S, Medina V, Kronish IM. Telemonitoring Adherence to Medications in Heart Failure Patients (TEAM-HF): A Pilot Randomized Clinical Trial. *J Card Fail.* 2017;23(4):345-349.
37. Giordano A, Scalvini S, Zanelli E, et al. Multicenter randomised trial on home-based telemanagement to prevent hospital readmission of patients with chronic heart failure. *Int J Cardiol.* 2009;131(2):192-199.
38. Goldberg LR, Piette JD, Walsh MN, et al. Randomized trial of a daily electronic home monitoring system in patients with advanced heart failure: the Weight Monitoring in Heart Failure (WHARF) trial. *Am Heart J.* 2003;146(4):705-712.
39. Goldman LE, Sarkar U, Kessell E, et al. Support from hospital to home for elders: a randomized trial. *Ann Intern Med.* 2014;161(7):472-481.
40. Graumlich JF, Novotny NL, Stephen Nace G, et al. Patient readmissions, emergency visits, and adverse events after software-assisted discharge from hospital: cluster randomized trial. *J Hosp Med.* 2009;4(7):E11-19.
41. Gurwitz JH, Field TS, Ogarek J, et al. An electronic health record-based intervention to increase follow-up office visits and decrease rehospitalization in older adults. *J Am Geriatr Soc.* 2014;62(5):865-871.
42. Haag JD, Davis AZ, Hoel RW, et al. Impact of Pharmacist-Provided Medication Therapy Management on Healthcare Quality and Utilization in Recently Discharged Elderly Patients. *Am Health Drug Benefits.* 2016;9(5):259-268.
43. Habib B, Buckeridge D, Bustillo M, et al. Smart About Meds (SAM): a pilot randomized controlled trial of a mobile application to improve medication adherence following hospital discharge. *JAMIA Open.* 2021;4(3):ooab050.

© 2025 Pattar BSB et al. *JAMA Network Open*.

44. Hajizadeh N, Polo J, Ordoñez K, et al. *Referral to Telehealth Delivered Pulmonary Rehabilitation (TelePR) Versus Standard Pulmonary Rehabilitation (SPR) in Hispanic and African Patients Hospitalized for COPD Exacerbations: Results of a Randomized Controlled Trial.* 2020.
45. Hale TM, Jethwani K, Kandola MS, Saldana F, Kvedar JC. A Remote Medication Monitoring System for Chronic Heart Failure Patients to Reduce Readmissions: A Two-Arm Randomized Pilot Study. *J Med Internet Res.* 2016;18(5):e91.
46. Heaton PC, Frede S, Kordahi A, et al. Improving care transitions through medication therapy management: A community partnership to reduce readmissions in multiple health-systems. *J Am Pharm Assoc (2003).* 2019;59(3):319-328.
47. Ho TW, Huang CT, Chiu HC, et al. Effectiveness of Telemonitoring in Patients with Chronic Obstructive Pulmonary Disease in Taiwan-A Randomized Controlled Trial. *Sci Rep.* 2016;6:23797.
48. Hsieh HL, Kao CW, Cheng SM, Chang YC. A Web-Based Integrated Management Program for Improving Medication Adherence and Quality of Life, and Reducing Readmission in Patients With Atrial Fibrillation: Randomized Controlled Trial. *J Med Internet Res.* 2021;23(9):e30107.
49. Indraratna P, Biswas U, McVeigh J, et al. A Smartphone-Based Model of Care to Support Patients With Cardiac Disease Transitioning From Hospital to the Community (TeleClinical Care): Pilot Randomized Controlled Trial. *JMIR Mhealth Uhealth.* 2022;10(2):e32554.
50. Jimenez S, Enjuanes C, Verdu-Rotellar JM, et al. Impact on clinical events and healthcare costs of adding telemedicine to multidisciplinary care of patients with heart failure and mid-range or preserved LVEF: a randomised controlled trial. *European Heart Journal.* 2017;38(suppl_1).
51. Johnson AE, Routh S, Taylor CN, et al. Developing and Implementing an mHealth Heart Failure Self-care Program to Reduce Readmissions: Randomized Controlled Trial. *JMIR Cardio.* 2022;6(1):e33286.
52. Kant N, Garssen SH, Vernooij CA, et al. Enhancing discharge decision-making through continuous monitoring in an acute admission ward: a randomized controlled trial. *Intern Emerg Med.* 2024;19(4):1051-1061.
53. Khonsari S, Subramanian P, Chinna K, Latif LA, Ling LW, Gholami O. Effect of a reminder system using an automated short message service on medication adherence following acute coronary syndrome. *Eur J Cardiovasc Nurs.* 2015;14(2):170-179.
54. Kotooka N, Kitakaze M, Nagashima K, et al. The first multicenter, randomized, controlled trial of home telemonitoring for Japanese patients with heart failure: home telemonitoring study for patients with heart failure (HOMES-HF). *Heart Vessels.* 2018;33(8):866-876.
55. Kowalkowski MA, Rios A, McSweeney J, et al. Effect of a Transitional Care Intervention on Rehospitalization and Mortality after Sepsis: A 12-Month Follow-up of a Randomized Clinical Trial. *Am J Respir Crit Care Med.* 2022;206(6):783-786.

© 2025 Pattar BSB et al. *JAMA Network Open*.

56. Kraai I, de Vries A, Vermeulen K, et al. The value of telemonitoring and ICT-guided disease management in heart failure: Results from the IN TOUCH study. *Int J Med Inform.* 2016;85(1):53-60.
57. Krzowski B, Boszko M, Peller M, et al. Mobile application and digital system for patients after myocardial infarction: early results from a randomized trial. *Pol Arch Intern Med.* 2023;133(9).
58. Kulshreshtha A, Kvedar JC, Goyal A, Halpern EF, Watson AJ. Use of remote monitoring to improve outcomes in patients with heart failure: a pilot trial. *Int J Telemed Appl.* 2010;2010:870959.
59. Lee TC, Kaiser TE, Alloway R, Woodle ES, Edwards MJ, Shah SA. Telemedicine Based Remote Home Monitoring After Liver Transplantation: Results of a Randomized Prospective Trial. *Ann Surg.* 2019;270(3):564-572.
60. Levine DM, Cueva MA, Shi S, et al. Skilled Nursing Facility Care at Home for Adults Discharged From the Hospital: A Pilot Randomized Controlled Trial. *Journal of Applied Gerontology.* 2022;41(6):1585-1594.
61. Levine DM, Paz M, Burke K, et al. Remote vs In-home Physician Visits for Hospital-Level Care at Home: A Randomized Clinical Trial. *JAMA Netw Open.* 2022;5(8):e2229067.
62. Liang HY, Hann Lin L, Yu Chang C, Mei Wu F, Yu S. Effectiveness of a Nurse-Led Tele-Homecare Program for Patients With Multiple Chronic Illnesses and a High Risk for Readmission: A Randomized Controlled Trial. *J Nurs Scholarsh.* 2021;53(2):161-170.
63. Lisby M, Bonnerup DK, Brock B, et al. Medication Review and Patient Outcomes in an Orthopedic Department: A Randomized Controlled Study. *J Patient Saf.* 2018;14(2):74-81.
64. López-Liria R, López-Villegas A, Enebakk T, Thunhaug H, Lappegård KT, Catalán-Matamoros D. Telemonitoring and Quality of Life in Patients after 12 Months Following a Pacemaker Implant: the Nordland Study, a Randomised Trial. *Int J Environ Res Public Health.* 2019;16(11).
65. Lyngå P, Persson H, Hägg-Martinell A, et al. Weight monitoring in patients with severe heart failure (WISH). A randomized controlled trial. *Eur J Heart Fail.* 2012;14(4):438-444.
66. Madigan E, Schmotzer BJ, Struk CJ, et al. Home health care with telemonitoring improves health status for older adults with heart failure. *Home Health Care Serv Q.* 2013;32(1):57-74.
67. McWilliams A, Roberge J, Anderson WE, et al. Aiming to Improve Readmissions Through InteGrated Hospital Transitions (AIRTIGHT): a Pragmatic Randomized Controlled Trial. *J Gen Intern Med.* 2019;34(1):58-64.
68. Mehta SJ, Hume E, Troxel AB, et al. Effect of Remote Monitoring on Discharge to Home, Return to Activity, and Rehospitalization After Hip and Knee Arthroplasty: A Randomized Clinical Trial. *JAMA Netw Open.* 2020;3(12):e2028328.
69. Mínguez P, Cadavid B, Mata C, et al. Early Assisted Discharge With Generic Telemedicine for Chronic Obstructive Pulmonary Disease Exacerbations: Results of a Randomized Controlled Trial. *CHEST.* 2014;145(3):198A.

© 2025 Pattar BSB et al. *JAMA Network Open*.

70. Mizukawa M, Moriyama M, Yamamoto H, et al. Nurse-Led Collaborative Management Using Telemonitoring Improves Quality of Life and Prevention of Rehospitalization in Patients with Heart Failure. *Int Heart J.* 2019;60(6):1293-1302.
71. Mousa AY, Broce M, Monnett S, Davis E, McKee B, Lucas BD. Results of Telehealth Electronic Monitoring for Post Discharge Complications and Surgical Site Infections following Arterial Revascularization with Groin Incision. *Ann Vasc Surg.* 2019;57:160-169.
72. Nipp RD, El-Jawahri A, Ruddy M, et al. Pilot randomized trial of an electronic symptom monitoring intervention for hospitalized patients with cancer. *Ann Oncol.* 2019;30(2):274-280.
73. Nipp RD, Horick NK, Qian CL, et al. Effect of a Symptom Monitoring Intervention for Patients Hospitalized With Advanced Cancer: A Randomized Clinical Trial. *JAMA Oncology.* 2022;8(4):571-578.
74. Noel K, Messina C, Hou W, Schoenfeld E, Kelly G. Tele-transitions of care (TTOC): a 12-month, randomized controlled trial evaluating the use of Telehealth to achieve triple aim objectives. *BMC Fam Pract.* 2020;21(1):27.
75. Ong MK, Romano PS, Edgington S, et al. Effectiveness of Remote Patient Monitoring After Discharge of Hospitalized Patients With Heart Failure: The Better Effectiveness After Transition -- Heart Failure (BEAT-HF) Randomized Clinical Trial. *JAMA Intern Med.* 2016;176(3):310-318.
76. Pietrantonio F, Vinci A, Rosiello F, et al. Green Line Hospital-Territory Study: A Single-Blind Randomized Clinical Trial for Evaluation of Technological Challenges of Continuous Wireless Monitoring in Internal Medicine, Preliminary Results. *Int J Environ Res Public Health.* 2021;18(19).
77. Pollak KI, Gao X, Beliveau J, Griffith B, Kennedy D, Casarett D. Pilot Study to Improve Goals of Care Conversations Among Hospitalists. *J Pain Symptom Manage.* 2019;58(5):864-870.
78. Polo J, Basile MJ, Zhang M, et al. Application of the RE-AIM framework to evaluate the implementation of telehealth pulmonary rehabilitation in a randomized controlled trial among African-American and Hispanic patients with advanced stage Chronic Obstructive Pulmonary Disease. *BMC Health Serv Res.* 2023;23(1):515.
79. Pooni A, Brar MS, Anpalagan T, et al. Home to Stay: A Randomized Controlled Trial Evaluating the Effect of a Postdischarge Mobile App to Reduce 30-Day Readmission Following Elective Colorectal Surgery. *Ann Surg.* 2023;277(5):e1056-e1062.
80. Prvu Bettger J, Green CL, Holmes DN, et al. Effects of Virtual Exercise Rehabilitation In-Home Therapy Compared with Traditional Care After Total Knee Arthroplasty: VERITAS, a Randomized Controlled Trial. *J Bone Joint Surg Am.* 2020;102(2):101-109.
81. Ravn-Nielsen LV, Duckert ML, Lund ML, et al. Effect of an In-Hospital Multifaceted Clinical Pharmacist Intervention on the Risk of Readmission: A Randomized Clinical Trial. *JAMA Intern Med.* 2018;178(3):375-382.
82. Riegel B, Carlson B, Kopp Z, LePetri B, Glaser D, Unger A. Effect of a standardized nurse case-management telephone intervention on resource use in patients with chronic heart failure. *Arch Intern Med.* 2002;162(6):705-712.

© 2025 Pattar BSB et al. *JAMA Network Open*.

83. Røsstad T, Salvesen Ø, Steinsbekk A, Grimsmo A, Sletvold O, Garåsen H. Generic care pathway for elderly patients in need of home care services after discharge from hospital: a cluster randomised controlled trial. *BMC Health Serv Res.* 2017;17(1):275.
84. Rubin DJ, Gogineni P, Deak A, et al. The Diabetes Transition of Hospital Care (DiaTOHC) Pilot Study: A Randomized Controlled Trial of an Intervention Designed to Reduce Readmission Risk of Adults with Diabetes. *J Clin Med.* 2022;11(6).
85. Saleh S, Skeie S, Grundt H. Re-admission and quality of life among patients with chronic obstructive pulmonary disease after telemedicine video nursing consultation - a randomized study. *Multidiscip Respir Med.* 2023;18(1):918.
86. Santana MJ, Holroyd-Leduc J, Southern DA, et al. A randomised controlled trial assessing the efficacy of an electronic discharge communication tool for preventing death or hospital readmission. *BMJ Qual Saf.* 2017;26(12):993-1003.
87. Schmaderer MS, Struwe L, Loecker C, et al. Mobile Health Self-management Interventions for Patients With Heart Failure: A Pilot Study. *J Cardiovasc Nurs.* 2022;37(5):E149-e159.
88. Schwarz KA, Mion LC, Hudock D, Litman G. Telemonitoring of heart failure patients and their caregivers: a pilot randomized controlled trial. *Prog Cardiovasc Nurs.* 2008;23(1):18-26.
89. Somsiri V, Asdornwised U, O'Connor M, Suwanugsorn S, Chansatitporn N. Effects of a Transitional Telehealth Program on Functional Status, Rehospitalization, and Satisfaction With Care in Thai Patients with Heart Failure. *Home Health Care Management & Practice.* 2021;33(2):72-80.
90. Soran OZ, Piña IL, Lamas GA, et al. A randomized clinical trial of the clinical effects of enhanced heart failure monitoring using a computer-based telephonic monitoring system in older minorities and women. *J Card Fail.* 2008;14(9):711-717.
91. Sørensen CA, de Thurah A, Lisby M, Olesen C, Sørensen SB, Enemark U. Cost-consequence analysis of self-administration of medication during hospitalization: a pragmatic randomized controlled trial in a Danish hospital setting. *Ther Adv Drug Saf.* 2020;11:2042098620929921.
92. Spaulding A, Loomis E, Brennan E, et al. Postsurgical Remote Patient Monitoring Outcomes and Perceptions: A Mixed-Methods Assessment. *Mayo Clin Proc Innov Qual Outcomes.* 2022;6(6):574-583.
93. Spierling Bagsic SR, Fortmann AL, San Diego ERN, et al. Outcomes of the Dulce Digital-COVID Aware (DD-CA) discharge texting platform for US/Mexico border Hispanic individuals with diabetes. *Diabetes Res Clin Pract.* 2024;210:111614.
94. Tamblyn R, Abrahamowicz M, Buckeridge DL, et al. Effect of an Electronic Medication Reconciliation Intervention on Adverse Drug Events: A Cluster Randomized Trial. *JAMA Netw Open.* 2019;2(9):e1910756.
95. Taylor SP, Murphy S, Rios A, et al. Effect of a Multicomponent Sepsis Transition and Recovery Program on Mortality and Readmissions After Sepsis: The Improving Morbidity During Post-Acute Care Transitions for Sepsis Randomized Clinical Trial. *Crit Care Med.* 2022;50(3):469-479.

© 2025 Pattar BSB et al. *JAMA Network Open*.

96. Tchalla A, Marchesseau D, Cardinaud N, et al. Effectiveness of a home-based telesurveillance program in reducing hospital readmissions in older patients with chronic disease: The eCOBAHLT randomized controlled trial. *J Telemed Telecare.* 2023:1357633x231174488.
97. Triller DM, Hamilton RA. Effect of pharmaceutical care services on outcomes for home care patients with heart failure. *Am J Health Syst Pharm.* 2007;64(21):2244-2249.
98. van der Storm SL, Consten ECJ, Govaert M, et al. Better stoma care using the Stoma App: does it help? A first randomized double-blind clinical trial on the effect of mobile healthcare on quality of life in stoma patients. *Surg Endosc.* 2024;38(3):1442-1453.
99. Vianello A, Fusello M, Gubian L, et al. Home telemonitoring for patients with acute exacerbation of chronic obstructive pulmonary disease: a randomized controlled trial. *BMC Pulm Med.* 2016;16(1):157.
100. Villani A, Malfatto G, Compare A, et al. Clinical and psychological telemonitoring and telecare of high risk heart failure patients. *J Telemed Telecare.* 2014;20(8):468-475.
101. Visade F, Lambeaux D, Delecluse C, et al. Implementation of a multi-interventional approach to improve medication safety in older hospitalized patients: Feasibility and impact on 30-day rehospitalization rate. *Ann Pharm Fr.* 2022;80(4):543-553.
102. Visperas AT, Greene KA, Krebs VE, Klika AK, Piuzzi NS, Higuera-Rueda CA. A Web-Based Interactive Patient-Provider Software Platform Does Not Increase Patient Satisfaction or Decrease Hospital Resource Utilization in Total Knee and Hip Arthroplasty Patients in a Single Large Hospital System. *J Arthroplasty.* 2021;36(7):2290-2296.e2291.
103. Volpp KG, Troxel AB, Mehta SJ, et al. Effect of Electronic Reminders, Financial Incentives, and Social Support on Outcomes After Myocardial Infarction: The HeartStrong Randomized Clinical Trial. *JAMA Intern Med.* 2017;177(8):1093-1101.
104. Wakefield BJ, Ward MM, Holman JE, et al. Evaluation of home telehealth following hospitalization for heart failure: a randomized trial. *Telemed J E Health.* 2008;14(8):753-761.
105. Weiss ME, Yakusheva O, Bobay KL, et al. Effect of Implementing Discharge Readiness Assessment in Adult Medical-Surgical Units on 30-Day Return to Hospital: The READI Randomized Clinical Trial. *JAMA Netw Open.* 2019;2(1):e187387.
106. Wen F, Du G, Tian F, Zhao X. Application and Effect of Patient Portal System in the Remote Management of Chronic Diseases for Older Adults With Coronary Heart Disease. *Sichuan Da Xue Xue Bao Yi Xue Ban.* 2024;55(2):418-424.
107. Widmer RJ, Allison TG, Lennon R, Lopez-Jimenez F, Lerman LO, Lerman A. Digital health intervention during cardiac rehabilitation: A randomized controlled trial. *Am Heart J.* 2017;188:65-72.
108. Wilson PM, Ramar P, Philpot LM, et al. Effect of an Artificial Intelligence Decision Support Tool on Palliative Care Referral in Hospitalized Patients: A Randomized Clinical Trial. *J Pain Symptom Manage.* 2023;66(1):24-32.

© 2025 Pattar BSB et al. *JAMA Network Open*.

109. Wolf A, Fors A, Ulin K, Thorn J, Swedberg K, Ekman I. An eHealth Diary and Symptom-Tracking Tool Combined With Person-Centered Care for Improving Self-Efficacy After a Diagnosis of Acute Coronary Syndrome: A Substudy of a Randomized Controlled Trial. *J Med Internet Res.* 2016;18(2):e40.
110. Woodend AK, Sherrard H, Fraser M, Stuewe L, Cheung T, Struthers C. Telehome monitoring in patients with cardiac disease who are at high risk of readmission. *Heart Lung.* 2008;37(1):36-45.
111. Yap DFS, Khairudin NA, Sabarudin NDA, Wong SW. Comparing service models: pharmacist-assisted transition of care (TOC) versus standard of care (SOC) towards effect on healthcare resource utilization among patients from medical wards. *Journal of Health and Translational Medicine.* 2022;25:18-26.
112. Yin H, Tan C, Li X, Wang J, Wu Z. Effect of continuous weight intervention on cardiac function and quality of life in patients with chronic congestive heart failure. *Chinese Nursing Research.* 2019;33(6):911-916.
113. You J, Wang S, Li J, Luo Y. Usefulness of a Nurse-Led Program of Care for Management of Patients with Chronic Heart Failure. *Med Sci Monit.* 2020;26:e920469.
114. Yu C, Liu C, Du J, et al. Smartphone-based application to improve medication adherence in patients after surgical coronary revascularization. *Am Heart J.* 2020;228:17-26.
115. Zhao Y. Effects of a discharge planning intervention for elderly patients with coronary heart disease in Tianjin, China: a randomized controlled trial. 2004.
116. Zisis G, Carrington MJ, Oldenburg B, et al. An m-Health intervention to improve education, self-management, and outcomes in patients admitted for acute decompensated heart failure: barriers to effective implementation. *European Heart Journal - Digital Health.* 2021;2(4):649-657.

© 2025 Pattar BSB et al. *JAMA Network Open*.
