# Supplementary material for: Electronic Health Record Interventions to Reduce Risk of Hospital Readmissions: A Systematic Review and Meta-Analysis
Source: JAMA Netw Open. 2025 Jul 17;8(7):e2521785. doi: 10.1001/jamanetworkopen.2025.21785 (PMC12272288; doi:10.1001/jamanetworkopen.2025.21785)
Supplement: Supplement 2. — Data Sharing Statement [file jamanetwopen-e2521785-s002.pdf]

Data Sharing Statement

Pattar. Electronic Health Record Interventions to Reduce Risk of Hospital Readmissions. *JAMA Netw Open*. Published July 17, 2025. doi:10.1001/jamanetworkopen.2025.21785

Data
Data available: No

Additional Information
Explanation for why data not available: Our data is secondary, from published resources, and thus we are unable to share the data from the included studies that is not already accessible.
